# Serious adverse drug reactions in sub‐Saharan Africa in the era of antiretroviral treatment: A systematic review

**DOI:** 10.1002/prp2.875

**Published:** 2021-11-05

**Authors:** Johannes P. Mouton, Nicole Jobanputra, Gayle Tatz, Karen Cohen

**Affiliations:** ^1^ Division of Clinical Pharmacology Department of Medicine University of Cape Town Cape Town South Africa

**Keywords:** drug safety, medicine safety, pharmacoepidemiology, systematic review

## Abstract

We aimed to summarize and describe the burden of serious adverse drug reactions (ADRs) in sub‐Saharan Africa (SSA) in the era of antiretroviral therapy. We searched Medline, CINAHL, Africa‐Wide Information, Scopus, and Web of Science, without language restriction up to March 2021. We hand‐searched reference lists, conference abstracts, and dissertation databases. We included studies reporting proportions of admissions attributed to ADRs, admissions prolonged by ADRs, or in‐hospital deaths attributed to ADRs. Two reviewers independently screened the studies, reviewed the study quality using a previously published tool, and extracted the data. We tested for heterogeneity using I^2^‐statistics and summarized the study results using medians and interquartile ranges. Subgroup analyses summarized the results by study quality, setting, methodology, and population. From 1005 unique references identified, we included 15 studies. Median study quality was 7/10; heterogeneity was very high. Median [IQR] proportion of admissions attributed to ADRs was 4.8% [1.5% to 7.0%] (14 studies) and 6.4% [4.0% to 8.4%] in nine active surveillance studies in adults. Two pediatric studies reported the proportion of admissions prolonged by ADRs (0.29% and 0.99%). Three studies reported the proportion of in‐hospital deaths attributed to ADRs (2.5%, 13%, and 16%). Antiretroviral and antituberculosis drugs were often implicated in serious ADRs. Evidence of the burden of serious ADRs in SSA is patchy and heterogeneous. A few high‐quality studies suggest that the burden is considerable, and that it reflects the regional impact of the HIV pandemic. Further characterization of this burden is required, ideally in studies of standardized methodology.

## 1 INTRODUCTION

The burden of adverse drug reactions (ADRs) in low‐ and middle‐income countries (LMICs) may differ from that in high‐income settings for a variety of reasons, including differences in disease burden, differences in drug utilization patterns, a potential lack of effective drug quality control, and the high risk for prescribing and dispensing errors that occur in overburdened healthcare systems. Previous systematic reviews summarizing the global burden of ADRs^1^, ^2^, ^3^, ^4^, ^5^, ^6^ included only a few surveys from LMICs, which limits the generalizability of their results to LMIC settings.

In sub‐Saharan Africa (SSA), an epidemiological transition is taking place, with high prevalence of both non‐communicable disease and infectious disease, particularly HIV. The World Health Organization (WHO) first introduced guidelines for scaling up antiretroviral therapy (ART) in resource‐limited settings in 2002.^7^ Large national ART programs in SSA could potentially contribute significantly to the burden of ADRs in this region.

Serious ADRs are those that result in death, are life‐threatening, result in hospital admission or prolong an existing hospital admission, result in persistent or significant disability or incapacity, or result in a congenital anomaly or cancer.^8^, ^9^ This systematic review aims to summarize and describe data on the burden of serious ADRs in SSA in the era of ART. We specifically focus on ADRs that cause hospital admission, prolong an existing hospital admission, or cause in‐hospital death, as these three categories of serious ADRs are the ones most frequently measured by surveys, are reasonably easy to verify, are not subjectively judged, and do not require longitudinal data.

The specific objectives of this systematic review are:

### 1.1 Primary objective

1. To summarize the proportions of hospital admissions attributable to ADRs, hospital admissions prolonged by ADRs, and in‐hospital deaths attributable to ADRs in SSA in the ART era.

### 1.2 Secondary objectives

1. To summarize the proportions of hospital admissions attributable to preventable ADRs, hospital admissions prolonged by preventable ADRs, and in‐hospital deaths attributable to preventable ADRs in SSA in the ART era.
2. To describe common clinical presentations of serious ADRs, and drugs commonly implicated in serious ADRs, in SSA in the ART era.
3. To explore the contribution of HIV and ART to the burden of serious ADRs in SSA in the ART era.
4. To explore methodological and quality issues in ADR surveys conducted in SSA in the ART era.

## 2 METHODS

### 2.1 Criteria for considering studies for this review

We reviewed observational studies from SSA, published since 2002 (the year of the first WHO ART guideline for resource‐constrained settings^7^), which reported any of the following proportions:

- the proportion of hospital admissions attributable to ADRs,
- the proportion of hospital admissions prolonged by ADRs, or
- the proportion of in‐hospital deaths attributable to ADRs.

Prospective or retrospective cohort studies, cross‐sectional studies, as well as data collected in the baseline survey / control arm of trials were eligible for inclusion. Forty‐eight countries, listed in the supplement, were defined as SSA countries in accordance with the World Bank's use of the term. We used original study authors’ definitions of “ADR”, “hospital”, and “admission”, but we specifically did not consider attendance at an emergency unit to be a hospital admission. We only included studies conducted in unselected hospital populations. No restrictions were applied in terms of publication language or type; we included studies available as abstracts only. No restrictions were applied in terms of study population age groups.

### 2.2 Search methods for identification of studies

We searched five databases (Medline, CINAHL, Africa‐Wide Information, Scopus, and Web of Science) for relevant journal articles.

The primary search strategy, developed with the help of a medical librarian, was based on a combination of free text and index term searches for searching Medline through EBSCOhost. Terms identifying SSA were derived from a recommendation by the University of North Carolina Libraries,^10^ and terms to identify ADRs were derived from a recommendation by the Cochrane Collaboration^11^ and from terms used in a previous systematic review.^12^ In addition to an “SSA concept” and an “ADR concept”, we included two more search concepts, relating to the “seriousness” and the “prevalence” of ADRs. Search strategies for all databases are included in the supplement.

Database searches were most recently conducted on 02 March 2021. Results were uploaded to an electronic deduplication and screening tool.

Database searches for journal articles were supplemented by hand‐searches conducted by one reviewer (JPM), including reference lists and lists of excluded studies of 30 previous review articles on various medicine safety topics,^1^, ^2^, ^3^, ^4^, ^5^, ^6^, ^12^, ^13^, ^14^, ^15^, ^16^, ^17^, ^18^, ^19^, ^20^, ^21^, ^22^, ^23^, ^24^, ^25^, ^26^, ^27^, ^28^, ^29^, ^30^, ^31^, ^32^, ^33^, ^34^, ^35^ reference lists of articles included in the current review, abstract books of the 2002 to 2020 annual meetings of the International Society of Pharmacovigilance and the International Society of Pharmacoepidemiology, and theses and dissertations via five databases, detailed in the supplement. Potentially relevant reports were added to the electronic deduplication and screening tool.

### 2.3 *Screening of title*/*abstracts*

After removing duplicates, two reviewers (JPM, and either NJ or GT) independently screened all reports on title and abstract. Reports were excluded if both reviewers agreed to exclude it; reasons for exclusion at this stage were not documented. Where no English‐language abstract was available, we used an online translator (Google Translate) to assess the potential relevance of the report.

### 2.4 *Obtaining full*‐*text articles*

We were able to obtain full‐texts of all the reports not excluded on title and abstract screening through the University of Cape Town Libraries; we did not need to contact authors for full‐texts. Where multiple reports were found to relate to one study, we combined the reports into one study at this point.

### 2.5 *Full*‐*text screening*

Full‐text studies were reviewed for inclusion independently by two reviewers (JPM, and NJ, GT, or KC). Studies in languages other than English were translated through an online translator (Google Translate). Disagreement over inclusion was resolved through discussion between the two reviewers, and a third reviewer could arbitrate. A reason for exclusion of the study was documented.

### 2.6 Quality assessment

Included studies were independently assessed for quality by two reviewers (JPM and GT) using a slightly modified version of a quality‐assessment tool developed specifically for ADR surveys.^12^ Our modification replaced the term ‘severity’ in the tool with the terms ‘seriousness or severity’. We calculated a quality score for each study as the total number of ‘yes’ responses out of tool's ten questions.

### 2.7 Data extraction

Two reviewers (JPM and GT) independently extracted data on study characteristics, the study setting, the study population, the study methodology, and study findings. (The data extraction form is included in the supplement). Disagreement was resolved through discussion between the two reviewers, and a third reviewer (KC) could arbitrate. We did not contact study authors with data extraction queries.

### 2.8 Data synthesis

Studies were grouped according to the data they contained relevant to this systematic review's three co‐primary objectives. Group 1 studies reported the proportion of hospital admissions attributed to ADRs, group 2 studies reported the proportion of hospital admissions prolonged by ADRs, and group 3 studies reported the proportion of in‐hospital deaths attributable to ADRs; a study could be included in more than one group. Since findings could be reported on the level of the patient or on the level of the admission (i.e., allowing for re‐admissions), we decided to use admission‐level data if reported, and patient‐level data if no admission‐level data were reported.

In the primary analysis, we pooled data from all studies in each group, regardless of study quality, study setting, methodological considerations, or study populations. We tested for heterogeneity using I^2^ statistics to decide whether to conduct meta‐analysis: in the presence of heterogeneity, we would summarize the proportions mentioned above as medians and interquartile ranges; in the absence of heterogeneity, we would conduct random‐effects meta‐analysis, calculating the pooled estimate proportion after Freeman‐Tukey double arcsine transformation to stabilize the variances.

We explored the proportions mentioned above by subgroups relating to study quality, study setting, methodological considerations, and study populations. Depending on heterogeneity within each subgroup (using I^2^ statistics) we would proceed to synthesize the data as above.

A similar approach was followed for the secondary objectives, where we summarized serious preventable ADRs. For the remainder of the secondary and explorative objectives, we narratively summarized clinical presentations commonly reported, drugs commonly implicated, and the contribution of HIV, ART, and methodological and quality issues.

## 3 RESULTS

**FIGURE 1:**
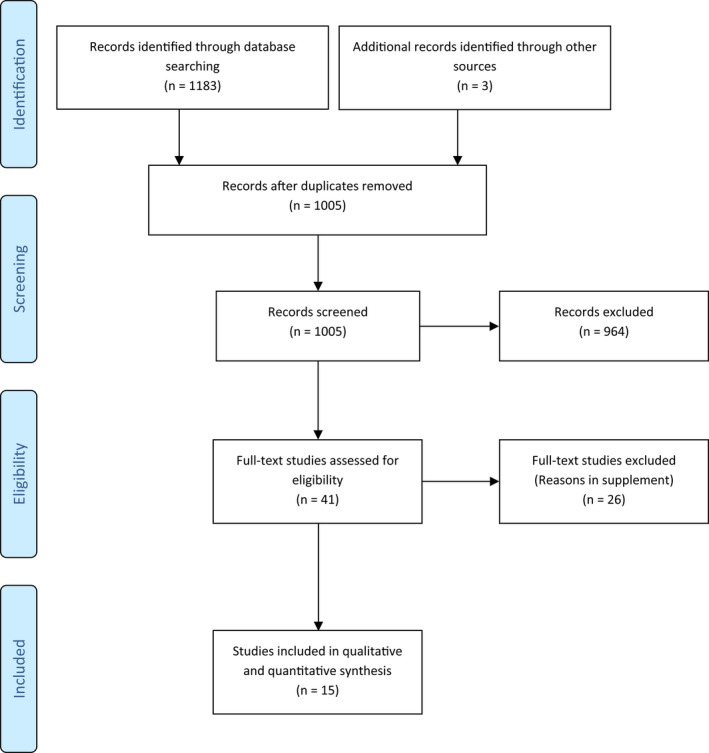
PRISMA diagram

Our electronic search yielded 1183 references and our hand search yielded three more; after deduplication 1005 references remained. We excluded 964 references as irrelevant on title/abstract screening and assessed 41 studies for eligibility on full text. We excluded 26, listed in the supplement with reasons. The most common reason for excluding studies on full text was that no disaggregated numerator was reported. These included studies that did not distinguish between serious and non‐serious ADRs, as well as studies of “umbrella topics”, such as drug‐related harm, which did not report ADRs separately. Fifteen studies^36^, ^37^, ^38^, ^39^, ^40^, ^41^, ^42^, ^43^, ^44^, ^45^, ^46^, ^47^, ^48^, ^49^, ^50^, ^51^, ^52^, ^53^ were included in this systematic review (Figure 1).

Quality scores ranged from 1/10 (in one study) to 10/10 (in four studies). Median [IQR] quality score was 7 [3 to 10]. All studies clearly reported the study design (Table 1), and most also clearly described data collection methods. In general, studies reported using standard methods for assessing causality, preventability, and seriousness/severity; however, details of the processes applying these methods (for example, the people performing the assessments, solving disagreements, etc.) were less well reported. The study characteristic reported least often was the process of establishing seriousness.

**TABLE 1:** Quality assessment of 15 studies included in the systematic review (adapted from Smyth et al^12^)

Quality question	Number (proportion) ‘yes’
01 Was study design clear?	15 (100%)
02 Were methods used to identify ADRs described in detail?	7 (47%)
03 Were data collection methods clearly described?	12 (80%)
04 Were the individuals who identified ADRs clearly described?	10 (67%)
05 Was the process of establishing the causal relationship described in detail?	8 (53%)
06 Were standard methods used in causality assessment?	13 (87%)
07 Was the process of establishing avoidability described in detail?	7 (47%)
08 Were standard methods used in avoidability assessment?	7 (47%)
09 Was the process of establishing seriousness or severity described in detail?	5 (33%)
10 Were standard methods used in seriousness or severity assessment?	8 (53%)

Table 2 summarizes the characteristics of studies included in this review.

**TABLE 2:** Characteristics of included studies

Study ID	Group*	Surveillance period and setting	Denominator	Surveillance methodology (identifying candidate ADRs)	ADR definition	Causality assessment	Seriousness assessment	Preventability assessment
Oshikoya (2007)^36^	1,2	36 months ending 2006, pediatric wards of single secondary/tertiary hospital in Nigeria	All pediatric patients admitted	Prospective and retrospective augmented folder review by multidisciplinary team	WHO definition,^54^ with specific inclusion: herbal / traditional medicines	Done according to Jones method.^55^ Numerator includes cases rated definite, probable, and possible	Implied (study reported serious outcomes)	Done. Criteria not reported
Mehta (2008)^37^	1,3	3 months in 2005, medical wards of single secondary/tertiary hospital in South Africa	Non‐random sample of adult patients (>16 years) admitted: 1% excluded for missing records. All deaths of adult patients admitted.	Prospective folder review by multidisciplinary team	WHO definition,^54^ with specific exclusions: intentional overdose and poor adherence; specific inclusion: accidental overdose	Done by multidisciplinary team (different from ADE surveillance team), according to WHO‐UMC method.^56^ Numerator includes cases rated definite, probable, and possible	Done by multidisciplinary team (different from ADE surveillance team), according to Temple criteria^57^	Done by multidisciplinary team (different from ADE surveillance team), according to Schumock criteria^58^
Soukho‐Kaya (2010)^38^, ^39^	1	12 months ending 2006, medical wards of single secondary/tertiary hospital in Mali	Non‐random sample of adult patients admitted: 4% excluded for receiving cancer chemotherapy	Prospective folder review	WHO definition^54^	Done according to French method.^59^ Numerator includes cases rated definite and probable.	Implied (study reported serious outcomes)	Not done
Oshikoya (2011)^40^	1	18 months ending 2007, pediatric wards of single secondary/tertiary hospital in Nigeria	Non‐random sample of pediatric patients admitted: unknown number excluded for admission <24 hours or repeat admission or missing records	Prospective augmented folder review by multidisciplinary team	Edwards and Aronson^8^	Done by same investigators who conducted ADE surveillance, according to Jones method.^55^ Not reported which categories were included in numerator.	Implied (study reported serious outcomes)	Done by same investigators who conducted ADE surveillance, according to Schumock criteria^58^
Tumwikirize (2011)^41^	1	6 months in 2005, medical wards of multiple hospitals (primary and secondary/tertiary) in Uganda	Non‐random sample of adult patients (>13 years) admitted: 35% excluded for no consent or too ill to cooperate	Prospective folder review by multidisciplinary team	WHO definition,^54^ with specific exclusion: herbal / traditional medicines	Done by multidisciplinary team (different from ADE surveillance team), according to Naranjo method.^60^ Numerator includes cases rated definite, probable, and possible	Implied (study reported serious outcomes)	Done according to Schumock criteria^58^
Kauffman (2014)^42^	1	6 months in 2012, single secondary/tertiary hospital in Malawi (wards not reported)	Non‐random sample of adult patients (>18 years) admitted: 84% excluded for missing records	Retrospective folder review	Not defined	Done by multidisciplinary team (different from ADE surveillance team), according to Naranjo method.^60^ Numerator includes cases rated definite, probable, and possible	Implied (study reported serious outcomes)	Not done
Aderemi‐Williams (2015)^43^	1	12 months ending 2009, medical wards of single secondary/tertiary hospital in Nigeria	Non‐random sample of adult patients admitted: 96% excluded for unclear reasons	Retrospective folder review	WHO definition,^54^ with specific exclusions: intentional overdose, accidental overdose, and poor adherence	Not done	Implied (study reported serious outcomes)	Not done
Ayetoro (2015)^44^, ^45^	1	12 months ending 2014, medical wards of single secondary/tertiary hospital in Nigeria	Not applicable	Spontaneous reporting	Not defined	Not reported or unclear	Implied (study reported serious outcomes)	Not reported or unclear
Mouton (2015)^46^	3	1 month in 2013, medical wards and intensive care units of multiple secondary/tertiary hospitals in South Africa	All deaths of adult patients admitted	Retrospective folder review by single investigator	Aronson and Ferner,^61^ with specific exclusion: intentional overdose	Done by multidisciplinary team (different from ADE surveillance team), according to WHO‐UMC method.^56^ Numerator includes cases rated definite, probable, and possible	Implied (study reported serious outcomes)	Done by multidisciplinary team (different from ADE surveillance team), according to Schumock criteria^58^
Mouton (2016)^47^	1	1 month in 2013, medical wards and intensive care units of multiple secondary/tertiary hospitals in South Africa	All admissions of adult patients	Prospective folder review by multidisciplinary team	Aronson and Ferner,^61^ with specific exclusions: intentional overdose and therapeutic failure	Done by multidisciplinary team (different from ADE surveillance team), according to WHO‐UMC method.^56^ Numerator includes cases rated definite, probable, and possible	Done by multidisciplinary team (different from ADE surveillance team), according to Temple criteria^57^	Done by multidisciplinary team (different from ADE surveillance team), according to Schumock criteria^58^
Russom (2017)^48^	1	5 months in 2014, all hospitals (primary and secondary/tertiary) in Eritrea (wards not reported)	Non‐random sample of adult and pediatric patients admitted: unknown number excluded for age <30 days or no consent or admitted for delivery	Prospective surveillance by multidisciplinary team.	WHO definition^54^	Done by investigators different from ADE surveillance team, according to Naranjo method.^60^ Numerator includes cases rated definite, probable, and possible	Done according to ICH/CIOMS criteria^9^	Done according to P‐method^62^
Angamo (2018)^49^, ^50^	1,3	16 months ending 2016, medical wards of single secondary/tertiary hospital in Ethiopia	Non‐random sample of adult patients (>18 years) admitted: 69% excluded for no consent or missing records or no drug exposure or not interviewed due to health or other reasons. All deaths of adult patients admitted.	Prospective augmented folder review by single investigator	WHO definition,^54^ with specific exclusions: intentional overdose, accidental overdose, drug abuse, and therapeutic failure	Done by multidisciplinary team (different from ADE surveillance team), according to Naranjo method.^60^ Numerator includes cases rated definite and probable	Done by same investigators who conducted ADE surveillance. Criteria not reported	Done. Criteria not reported
Makiwane (2019)^51^	1	3 months in 2016, pediatric wards of single secondary/tertiary hospital in South Africa	Non‐random sample of pediatric patients (<16 years) admitted: unknown number excluded for admission <24 hours or no consent	Prospective folder review by single investigator	WHO definition,^54^ with specific inclusion: herbal / traditional medicines	Done according to Naranjo method.^60^ Numerator includes cases rated definite, probable, and possible	Done according to ICH/CIOMS criteria^9^	Not done
Adedapo (2020)^52^	1	12 months ending 2013, medical wards of single secondary/tertiary hospital in Nigeria	Non‐random sample of adult patients admitted: 57% excluded for no consent or existing admissions or repeat admissions or very ill	Prospective augmented folder review	WHO definition,^54^ with specific inclusions: herbal / traditional medicines, medication errors	Done according to WHO‐UMC method.^56^ Numerator includes cases rated definite, probable, and possible	Implied (study reported serious outcomes)	Done according to Wolfe criteria^35^
Mouton (2020)^53^	1,2	1 month in 2015, pediatric wards and intensive care units of multiple secondary/tertiary hospitals in South Africa	Non‐random sample of admissions of pediatric patients: unknown number excluded for elective admissions, rehydration therapy, postnatal stays	Prospective and retrospective folder review by multidisciplinary team	Aronson and Ferner,^61^ with specific exclusions: herbal / traditional medicines, intentional overdose, poor adherence, medication errors without harm, therapeutic failure	Done by multidisciplinary team (different from ADE surveillance team), according to WHO‐UMC method.^56^ Numerator includes cases rated definite, probable, and possible	Done by multidisciplinary team (different from ADE surveillance team), according to Temple criteria^57^	Done by multidisciplinary team (different from ADE surveillance team), according to Schumock criteria^58^

* Group 1 studies report proportion of admissions attributed to ADRs. Group 2 studies report the proportion of admissions prolonged by ADRs. Group 3 studies report the proportion of in‐hospital deaths attributed to ADRs.

### 3.1 Group 1 studies

**FIGURE 2:**
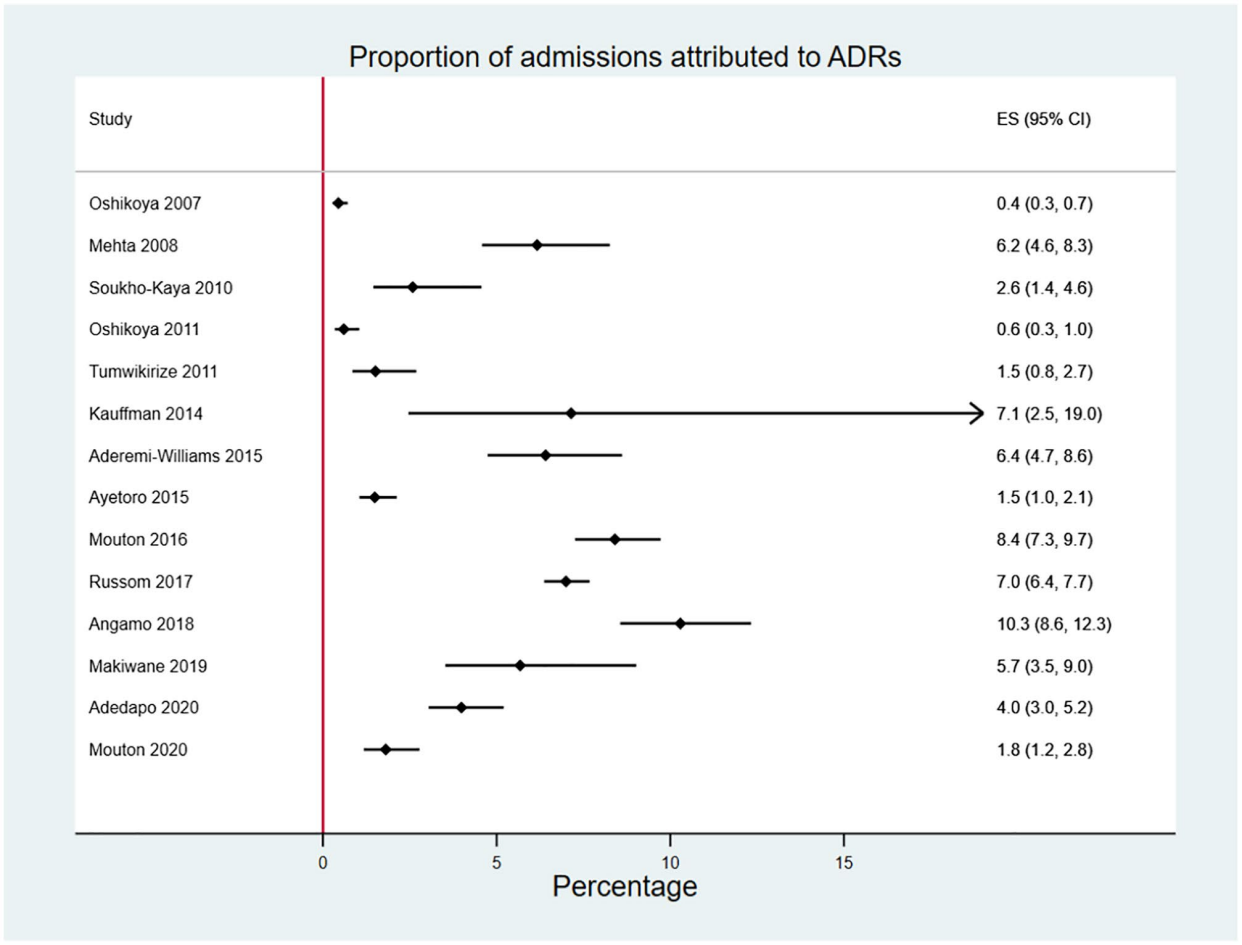
Forest plot of studies reporting proportion of admissions attributed to ADRs

Fourteen studies^36^, ^37^, ^38^, ^39^, ^40^, ^41^, ^42^, ^43^, ^44^, ^45^, ^47^, ^48^, ^49^, ^50^, ^51^, ^52^, ^53^ reported as outcome the proportion of admissions attributed to ADRs. To estimate the summary proportion of admissions attributed to ADRs, we first pooled all group 1 studies and tested for heterogeneity. Since very high heterogeneity was found (I^2^ = 98.2%), no meta‐analysis was performed. The median [IQR] proportion of admissions attributed to ADRs among the 14 studies was 4.8% [1.5% to 7.0%] (Figure 2).

We investigated heterogeneity among subgroups based on study quality, study setting, study methodology, and study population. Since there was very high heterogeneity in each subgroup, no meta‐analysis by subgroup was performed. Heterogeneity and summary proportions, by subgroup, are presented in the supplement.

One study, reported only in two conference abstracts,^44^, ^45^ was a low‐quality report of a spontaneous ADR reporting system in the medical wards of a Nigerian hospital, where 30/2012 (1.5%) patients were reported to have had an ADR‐related admission.

The remaining 13 studies were all conducted as active surveillance studies, eight of which were in adults,^37^, ^38^, ^39^, ^41^, ^42^, ^43^, ^47^, ^49^, ^50^, ^52^ four in children,^36^, ^40^, ^51^, ^53^ and one in a mixed adult and pediatric population.^48^ As this latter study presented data for adults and children separately, we could extract separate adult and pediatric datasets from it. Key results from Group 1 studies, grouped by the ADR detection method (spontaneous reporting vs. active surveillance) and study population (adults vs. children) are presented in Table 3.

**TABLE 3:** Key results from studies reporting the proportion of admissions attributed to ADRs (Group 1 studies). Studies grouped by ADR detection method and population studied

Study	Proportion of admissions attributed to ADRs / Proportion of patients with admissions attributed to ADRs	Most common clinical presentations of ADRs (n)	Most commonly implicated drugs / classes (n)	Proportion of patients with admissions attributed to ADRs who are PLWH
Spontaneous Reporting				
Ayetoro (2015)^44^, ^45^	30/2012 (1.5%) patients.	NR	NR	NR
Active Surveillance, Adults				
Mehta (2008)^37^	41/665 (6.2%) patients.	Metabolic (16), endocrine (10), hepatic (8), and neuropsychiatric (8)	Cardiovascular (22), antiretrovirals (17), oral hypoglycemic agents (7), non‐steroidal anti‐inflammatories (7)	38% PLWH
Soukho‐Kaya (2010)^38^, ^39^	11/426 (2.6%) patients.	Hypoglycemia (5)	NR	NR
Tumwikirize (2011)^41^	11/728 (1.5%) patients.	NR	NR	NR
Kauffman (2014)^42^	3/42 (7.1%) patients.	Anemia (1), hyperlactatemia (1), GIT distress (1)	Stavudine (2), metronidazole (1)	3/3 PLWH
Aderemi‐Williams (2015)^43^	40/624 (6.4%) patients.	Not clear	Not clear	NR
Mouton (2016)^47^	164/1951 (8.4%) admissions.	Renal impairment (24), hypoglycemia (22), DILI (20), hemorrhage (19), blood dyscrasias (14)	Rifampicin (17), enalapril (13), insulin (14), tenofovir (14), warfarin (13)	64/164 (38%) in PLWH
Russom (2017)^48^ (adults)	295/3415 (8.6%) patients.	NR	NR	NR
Angamo (2018)^49^, ^50^	103/1001 (10%) patients.	Hepatotoxicity (35), acute kidney injury (27), skin reactions (8), hypokalemia (7), gastrointestinal bleed/gastritis (7)	Isoniazid (23), furosemide (19), pyrazinamide (18) tenofovir (9), acetylsalicylic acid (9)	29/103 (28%) PLWH and on ART
Adedapo (2020)^52^	51/1280 (4.0%) patients.	NR	NR	NR
Active surveillance, children				
Oshikoya (2007)^36^	17/3821 (0.44%) patients.	Erythema multiforme (12)	Ampicillin (7), sulfadoxine / pyrimethamine (5), co‐trimoxazole (5), phenobarbitone (3), herbs (2)	NR
Oshikoya (2011)^40^	12/2004 (0.60%) patients.	Erythema multiforme (5), Stevens‐Johnson syndrome (2), macular and morbidiform rash (2)	Cotrimoxazole (6), ampicillin (4), sulfadoxine / pyrimethamine (3)	NR
Russom (2017)^48^ (children)	114/2433 (4.7%) patients.	NR	NR	NR
Makiwane (2019)^51^	16/282 (5.7%) patients.	NR	NR	NR
Mouton (2020)^53^	20/1106 (1.8%) admissions.	Urticaria (2), dystonia (2)	Prednisone (2), metoclopramide (2)	3/20 (15%) PLWH

Abbreviations: NR, not reported; PLWH, people living with HIV.

#### 3.1.1 Adult active surveillance studies

Nine active surveillance studies contributed adult data (Table 3). All were available as English full‐text reports, except one Malian study reported in both French‐language article^39^ and French‐language thesis^38^ with English abstracts. An Ethiopian study was reported in two complementary articles.^49^, ^50^ Median quality score was 7/10, ranging from 2/10 to 10/10. Studies were conducted in South Africa (two studies,^37^, ^47^ both high quality), Nigeria (two studies^43^, ^52^), and in Eritrea,^48^ Ethiopia,^49^, ^50^ Malawi,^42^ Mali,^38^, ^39^ and Uganda.^41^ Study duration ranged from 1 month to 16 months, for a median of 6 months. Six studies were single‐center studies at secondary‐ / tertiary‐level hospitals,^37^, ^38^, ^39^, ^42^, ^43^, ^49^, ^50^, ^52^ while three were conducted in multiple hospitals,^41^, ^47^, ^48^ including primary‐level hospitals in two.^41^, ^48^ Where reported, all studies were conducted in medical wards,^37^, ^38^, ^39^, ^41^, ^43^, ^47^, ^49^, ^50^, ^52^ additionally including intensive care units in one.^47^ Only one study reported universal sampling of all patients admitted to the study wards^47^; non‐random sampling of admitted patients was described in the remainder. In these studies, large numbers of potential participants were often excluded (35%,^41^ 57%,^52^ 69%,^49^, ^50^ 84%,^42^ and 96%^43^ in five studies, but only 1%^37^ and 4%^38^, ^39^ in two others). Reasons for excluding potential participants included no consent,^41^, ^48^, ^49^, ^50^, ^52^ missing records,^37^, ^42^, ^49^, ^50^ being too ill to cooperate,^41^, ^49^, ^50^, ^52^ admissions for cancer chemotherapy^38^, ^39^ or delivery,^48^ readmissions,^52^ no drug exposure,^49^, ^50^ or unclear reasons.^43^

Most studies^37^, ^38^, ^39^, ^41^, ^47^, ^48^, ^49^, ^50^, ^52^ used prospective folder review as surveillance methodology, but only four^37^, ^41^, ^47^, ^48^ reported this to have been conducted by multidisciplinary team. ADRs were mostly defined according to the WHO definition.^37^, ^38^, ^39^, ^41^, ^43^, ^48^, ^49^, ^50^, ^52^ Five studies^37^, ^41^, ^42^, ^47^, ^49^, ^50^ reported a clear two‐step method, with causality assessment conducted by a multidisciplinary team other than the surveillance team. Naranjo's causality assessment method (in 4 studies^41^, ^42^, ^48^, ^49^, ^50^) and the WHO‐UMC method (in three studies^37^, ^47^, ^52^) were mostly used. Six studies reported assessing the preventability of ADRs, using Schumock and Thornton criteria in three studies^37^, ^41^, ^47^ and unreported or other methods in three studies.^48^, ^49^, ^50^, ^52^

Apart from summary demographic statistics, the population included in studies’ denominator was generally poorly described. Mean or median age ranged from 36 to 50 years (reported in six studies^37^, ^38^, ^39^, ^41^, ^47^, ^49^, ^50^, ^52^), and the proportion of females from 42% to 56% (reported in seven studies^37^, ^38^, ^39^, ^41^, ^43^, ^47^, ^49^, ^50^, ^52^). Only two studies reported the proportion of patients included in the denominator that were exposed to drugs before their admission.^47^, ^49^, ^50^ Only two studies described the most common reason for admission, being malaria^41^ and cardiovascular disease^47^ respectively. HIV prevalence among patients included in the denominator was only reported in the South African studies, being 32% in the earlier^37^ and 29% in the later^47^ study. The proportion of patients in the denominator who were taking ART increased in the interval between these two studies from 5.2%^37^ to 14%,^47^ and was 11% in the Ethiopian study.^49^, ^50^

Among the nine active surveillance studies in adult populations, the median [IQR] proportion of admissions attributed to ADRs was 6.4% [4.0% to 8.4%]. Although more studies assessed the preventability of ADRs, only one study, from South Africa, reported the proportion of admissions attributed to preventable ADRs, which was 3.7%.^47^

Four studies reported the drugs or drug classes implicated in ADRs causing admission to hospital,^37^, ^42^, ^47^, ^49^, ^50^ with striking similarities: antiretroviral agents, antituberculosis therapy, cardiovascular drugs, and hypoglycemic drugs predominated. Common clinical presentations of these ADRs, as reported in five studies,^37^, ^38^, ^39^, ^42^, ^47^, ^49^, ^50^ mostly related to their hepatotoxic and nephrotoxic effects, and hypoglycemia. Bleeds from non‐steroidal anti‐inflammatories and antithrombotic agents were less frequently reported.^47^, ^49^, ^50^

Four studies reported HIV prevalence among patients who were admitted to hospital for ADRs. This was 38% in both South African studies,^37^, ^47^ 28% in the Ethiopian study,^49^, ^50^ and 3/3 in the Malawian study.^42^

#### 3.1.2 Pediatric active surveillance studies

Five active surveillance studies contributed pediatric data (Table 3), including two from Nigeria,^36^, ^40^ two from South Africa,^51^, ^53^ and one from Eritrea.^48^ All were available as English full‐text articles, with median quality score 5/10, although two studies^40^, ^53^ scored 10/10. Study duration ranged from 1 month to 36 months. Three studies were single‐center studies at secondary‐ / tertiary‐level hospitals^36^, ^40^, ^51^ while two were conducted in multiple hospitals,^48^, ^53^ including primary‐level hospitals in one.^48^ One study^53^ surveyed patients admitted to intensive care units in addition to those admitted to pediatric wards. Only one study reported universal sampling of all patients admitted to the study wards^36^; non‐random sampling of admitted patients was described in the remainder. In these studies, unknown numbers of potential participants were excluded for reasons including no consent,^48^, ^51^ missing records,^40^ short duration admissions,^40^, ^51^ admissions for rehydration,^53^ repeat admissions,^40^ elective admissions,^53^ or neonatal admissions.^48^, ^53^

Four studies reported that multidisciplinary teams conducted the surveillance.^36^, ^40^, ^48^, ^53^ Three studies^36^, ^40^, ^48^ defined ADRs according to the WHO definition. All studies included an assessment of causality, and four^36^, ^40^, ^48^, ^53^ included an assessment of preventability, although precise methods varied.

Only one study reported the proportion of patients included in the denominator that were exposed to drugs before their admission.^53^ Infectious diseases were frequently reported as the reason for admission.^36^, ^40^, ^51^, ^53^ The proportion of children in the denominator population who had HIV infection was only reported in the two South African studies.^51^, ^53^

The proportion of admissions attributed to ADRs ranged from 0.4% to 5.7%, median [IQR] 1.8% [0.6% to 4.7%]. At the low end of the range were the two large Nigerian studies which seemingly included older children, with one study reporting the mean age among all admissions being 6.4 years.^40^ In contrast, the South African studies’ populations were younger (median age 1.4 years^51^ and 0.9 years^53^). Hypersensitivity reactions, including erythema multiforme, Stevens Johnson syndrome, urticaria, and rashes were reported as the most common presentations, and antimicrobial medicines were most commonly implicated. Two studies reported the proportion of admissions attributed to preventable ADRs, which were 0.1%^40^ and 0.5%^53^ respectively. Only one study, conducted in South Africa, reported HIV prevalence among children admitted for ADRs, being 15%.^53^

### 3.2 Group 2 studies

We found no study conducted in adult populations reporting the proportion of hospital admissions prolonged by ADRs. Two pediatric studies, one from Nigeria^36^ and one from South Africa^53^ reported this proportion (Table 4). In the Nigerian study,^36^ 0.29% children experienced ADR‐related prolongation of their hospital admission. ADR preventability, presentations, implicated drugs, and HIV exposure were not reported. In the South African study^53^ 0.99% admissions were prolonged by ADRs. Most prolongations were for antibiotic‐associated diarrhea, although a variety of other admission‐prolonging ADRs occurred, including ADRs attributed to corticosteroids and immunosuppressants. Only one study reported the proportion of admissions prolonged by preventable ADRs, being 2/1106 (0.19%).^53^ Only one study, conducted in South Africa, reported HIV prevalence among children whose hospital stays were prolonged by ADRs, being 18%.^53^

**TABLE 4:** Key results from studies reporting the proportion of admissions prolonged by ADRs (Group 2 studies)

Study	Proportion of admissions prolonged by ADRs / Proportion of patients with admissions prolonged by ADRs	Most common clinical presentations of ADRs (n)	Most commonly implicated drugs / classes (n)	Proportion of patients with admissions prolonged by ADRs who are PLWH
Oshikoya (2007)^36^	11/3821 (0.29%) patients.	NR	NR	NR
Mouton (2020)^53^	11/1106 (0.99%) admissions.	Diarrhea (4), bicytopaenia (2)	Prednisone (3), methylprednisolone (3), tacrolimus (2), mycophenolic acid (2), amoxicillin (2)	2/11 (18%) PLWH

Abbreviations: NR, not reported; PLWH, people living with HIV.

### 3.3 Group 3 studies

Three high‐quality adult studies, two from South Africa^37^, ^46^ and one from Ethiopia,^49^, ^50^ reported the proportion of in‐hospital deaths to which ADRs contributed (Table 5). All three studies were conducted among adults in secondary / tertiary hospitals, with one^46^ also including patients in the intensive care unit. In two studies^46^, ^49^, ^50^ the proportion of deaths attributed to ADRs was the primary outcome measure, while the third^37^ mentioned the proportion of deaths attributed to ADRs as an additional outcome. The Ethiopian survey^49^, ^50^ only considered deaths from ADRs already present at time of admission, and not deaths from ADRs that developed during the hospital stay, and thus may have underestimated the deaths attributable to ADRs.

**TABLE 5:** Key results from studies reporting the proportion of in‐hospital deaths attributed to ADRs (Group 3 studies)

Study	Proportion of in‐hospital deaths attributed to ADRs	Most common clinical presentations of ADRs (n)	Most commonly implicated drugs / classes (n)	Proportion of people whose in‐hospital deaths were attributed to ADRs who were PLWH
Mehta (2008)^37^	2/80 (2.5%) deaths.	Acute renal failure (1), intracranial bleed (1)	Gentamycin (1), warfarin (1)	NR
Mouton (2015)^46^	56/357 (16%) deaths.	Renal failure (23), drug‐induced liver injury (10)	Tenofovir (14), rifampicin (9), co‐trimoxazole (7), furosemide (5), insulin (4)	31/56 (55%) PLWH
Angamo (2018)^49^, ^50^	15/116 (13%) deaths.	Hepatotoxicity (7), kidney injury (4)	Isoniazid (6), pyrazinamide (3), tenofovir (2), efavirenz (2), enalapril (2), furosemide (2)	6/15 (40%) PLWH

Abbreviations: NR, not reported; PLWH, people living with HIV.

The three studies respectively found 2/80 (2.5%),^37^ 56/357 (16%),^46^ and 15/116 (13%)^49^, ^50^ deaths were ADR‐related.

The proportion of deaths attributed to preventable ADRs was reported in two studies: 28/357 (7.8%) in South Africa^46^ and 14/116 (12%) in Ethiopia^49^, ^50^ respectively.

These two studies also reported the proportion of ADR‐related deaths in which decedents were people living with HIV (PLWH): 31/56 (55%)^46^ and 7/15 (47%)^49^, ^50^ respectively. In both studies, renal failure and drug‐induced liver injury were the most common ADRs resulting in death, and both studies listed antiretrovirals and antituberculosis drugs as the drugs most commonly implicated in ADR‐related deaths.

Multivariable logistic regression in one study^46^ identified HIV‐infection with antiretroviral treatment, higher drug count, and higher comorbidity score as independent risk factors for ADR‐related death. Unadjusted bivariate analyses in the other^49^, ^50^ also showed associations between ADR‐related death and exposure to antiretroviral treatment, higher drug count, and higher comorbidity score, as well as pre‐existing liver disease, a history of prior ADR, low body‐mass index, and exposure to antituberculosis drugs.

In addition to the three studies included in this group, fatal ADR outcomes were reported by nine other studies included in this review.^36^, ^38^, ^39^, ^40^, ^42^, ^44^, ^45^, ^48^, ^51^, ^52^, ^53^ However, these nine studies did not report the proportion of ADR‐related deaths against a denominator of all in‐hospital deaths.

A Nigerian study^52^ reported seven deaths attributed to ADRs among 67 adults with serious and non‐serious ADRs. These included three cases of Stevens Johnson Syndrome or toxic epidermal necrolysis with co‐trimoxazole and phenytoin, two cases of hemorrhage with heparin and diclofenac, and two cases of hepatotoxicity with anti‐tuberculosis therapy and herbal medicine. Two of the deaths occurred in PLWH. Among other adult studies, the Malawian study^42^ reported one fatal outcome (hyperlactatemia with stavudine) among their three patients with serious ADRs; the Malian study^38^, ^39^ reported three fatal outcomes (hypoglycemia, and “colchicine‐induced vomiting”) among 39 patients with 47 serious and non‐serious ADRs; and the spontaneous reporting study^44^, ^45^ reported no fatal outcomes among 30 patients with serious ADRs.

Pediatric studies generally reported low absolute numbers of fatalities. However, fatal outcomes were reported to occur in a relatively high proportion of serious ADRs: 2/17 serious ADRs were fatal (Stevens‐Johnson syndrome and hepatotoxicity) in one Nigerian study,^36^ 2/12 (Stevens‐Johnson syndrome) in the other,^40^ and 1/40 in a South African study.^53^ No fatal outcomes were reported among 61 serious and non‐serious ADRs in the other South African pediatric study.^51^

In the country‐wide Eritrean survey 48 fatal ADRs were reported among 5,848 patients admitted.^48^ The two most common ADRs resulting in death were anemia (attributed to various drugs, including zidovudine) and hepatotoxicity (mostly attributed to antituberculosis therapy). Drugs used in the management of HIV, TB, and opportunistic infections appear to have been implicated in 17 deaths.

## 4 DISCUSSION

A small number of relatively high‐quality studies report that serious ADRs contribute significantly to the burden of morbidity and mortality in SSA hospitals. Fourteen studies included in this review reported that a median 4.8% (IQR 1.5% to 7.0%) of admissions were attributed to ADRs; three studies reported that between 2.5% and 16% of in‐hospital deaths were attributed to ADRs.

This systematic review demonstrated the paucity of drug safety data from hospital settings in sub‐Saharan Africa, and echoes the paucity of drug safety data contributed from Africa to the global spontaneous reporting database described by others.^63^ Only 15 studies fulfilled the inclusion criteria, despite the fact that we searched databases focused on Africa as well as grey literature, and despite searching without language restrictions. Even given the overall low number of studies included, there were regional and population differences: two‐thirds of the included studies came from just two countries, South Africa and Nigeria, and none from a Central African country; few studies were conducted in pediatric settings, and none specifically in older adults. These are populations to be covered in future ADR surveillance activities.

Study quality, as measured by the tool we applied, was generally high. Nevertheless, to fully comprehend the context within which ADRs occur, and the risk for their occurrence, it is essential to understand the denominator study population, including their diseases and their drug exposures.^64^ Many studies included in this review did not clearly describe the denominator study population, and had this been included as a factor in the quality assessment, we may have reported lower quality overall. A further quality concern among nearly all studies included in this review is the high proportion of potential participants excluded from the surveillance activity. Non‐participation was often over 50% and as high as 96% in one study, which may have resulted in a biased sample. Again, although this is a serious quality concern, it is not reflected in the quality assessment tool we used in this review.

We found very high heterogeneity among the study results, which can be attributed to high variability in study designs as described in Table 2, and also to high variability in the study settings and populations, potentially including some unreported differences. It is well‐established that the reported prevalence of medication‐related hospital admissions depends on the setting, studied population, specific outcome investigated, and surveillance method.^30^ Unfortunately, calls for greater standardization in the methodology of medicine safety studies^8^, ^61^, ^64^ have largely gone unheeded.

Because of this high heterogeneity we did not conduct meta‐analysis of studies, but reported a median 4.8% (IQR 1.5% to 7.0%) as the proportion of admissions attributed to ADRs. This estimate agrees with the results of six earlier systematic reviews of ADR‐related admissions,^1^, ^2^, ^3^, ^4^, ^5^, ^6^ which estimated the proportion of admissions attributable to ADRs to range between 3.1% and 6.3% (some in sub‐group analyses).

A 2018 systematic review of African studies of adverse drug events or medication errors in hospitals^33^ differed from the current review as it included only peer‐reviewed publications, included studies from North Africa and studies predating the ART era, and only distinguished between serious and non‐serious events in some ADE cases. Moreover, despite defining ADRs as a subset of ADEs, the authors of that systematic review reported “overlooking” this factor,^33^ in the end pooling studies reporting an outcome of ADRs together with those reporting an outcome of ADEs. In view of the methodological differences between the two systematic reviews, it is not surprising that there is little overlap in the studies included, and a very different result. For the comparable outcome we described in our Group 1 studies (proportion of admissions attributable to ADRs, n = 14 studies, median proportion 4.8%) and the earlier review's outcome of proportion of admissions as a direct result of ADEs (n = 11 studies, median proportion 2.8%),^33^ only six studies were included in both reviews.

Another previous systematic review, published in 2016, aimed to compare adult ADR burdens in high‐income countries and LMICs.^16^ The authors found the median proportion of admissions attributed to ADRs to be lower in LMICs (5.5%) than in high‐income countries (6.3%).^16^ However, this review included only three studies from SSA among the 13 LMIC studies.^16^ Our comparable estimate (median 6.4% among nine adult active surveillance studies) is probably more representative, and read together with the results from other systematic reviews mentioned above^1^, ^2^, ^3^, ^4^, ^5^, ^6^ probably dispels the idea that the burden of ADR‐related admissions in SSA is lower than in high‐income countries.

The proportion of in‐hospital deaths attributed to ADRs in Europe was the topic of a 2021 systematic review. Six studies contributed 657 drug‐related deaths out of 7578 in‐hospital deaths, with the meta‐analytic estimated proportion being 7.3% (95% CI 4.1% to 12.5%).^65^ This estimate appears to agree with an earlier population‐based study using linked databases in Sweden, in which 6.4% of in‐hospital deaths were attributed to ADRs.^66^ We identified only three studies conducted in SSA to report this particular outcome, and these reported 2.5%, 13%, and 16% of deaths in adult medical wards were attributed to ADRs. The low number of SSA studies reporting this proportion as outcome precludes any meaningful interpretation of this proportion. However, it is notable that in two of the studies a large proportion of the deaths were associated with preventable ADRs. In addition, it is notable that most deaths were due to renal and liver injuries, with ART and antituberculosis therapy most often implicated. This stands in contrast with studies from Europe, which found hemorrhages to be most common fatal ADRs.^65^, ^66^

For several of our study objectives, we found limited data. Only two studies reported on admissions prolonged by ADRs, and there were minimal reports on admissions caused by or prolonged by preventable ADRs. These are knowledge gaps to be filled by future research.

Another exploratory objective yielding little data was describing the role played by HIV and ART in the serious ADR burden in SSA. Although rarely reported, it was consistently reported that HIV prevalence among patients with serious ADRs was higher than HIV prevalence among the denominator population.^37^, ^46^, ^47^, ^49^, ^50^, ^53^ This imbalance was most pronounced for the outcome of ADR‐related deaths. However, this finding should be interpreted with care. Where antiretroviral therapy was implicated in causing ADRs, these were often older antiretrovirals, including stavudine and efavirenz, and the ADRs were often unpredictable and unavoidable events. ART programs in SSA are continuously improving and introducing newer drugs with fewer toxicities: stavudine has been phased out as first‐line ART option in the period 2006 to 2011,^67^ and efavirenz is currently being phased out in favor of dolutegravir. Tenofovir alafenamide has better renal safety than tenofovir disoproxil fumarate^68^ and may in future replace it in ART programs.

## 5 CONCLUSION

We have shown that evidence of the burden of serious ADRs in SSA is patchy and highly heterogeneous. Nevertheless, a few high‐quality studies suggest that the burden is considerable. A unique feature of the ADR burden in this region is the frequency at which people living with HIV appear affected, and the frequency with which ART and medicines used in the management of opportunistic infections are implicated in this burden. However, the risk of ADRs should be considered against the risks associated with non‐treatment,^64^ and there is no doubt that the benefit of these medicines massively outweigh their risk of harm. Further characterization of the serious ADR burden in SSA is required, particularly in pediatric and elderly populations, and in countries other than South Africa and Nigeria. This should ideally be performed by conducting studies of standardized methodology. As a first step toward limiting the risk of harm caused by medicines, the focus should be on avoiding preventable ADRs, which are often caused by errors. Investing in support systems that catch errors before harm occurs is required, particularly in under‐resourced and overburdened health care settings such as those in SSA.

## CONFLICT OF INTEREST

The authors have no conflicts of interest to declare.

## AUTHOR CONTRIBUTIONS

JPM and KC conceived and designed the study. JPM conducted the searches. JPM, NJ, GT, and KC screened studies for inclusion. JPM, GT, and KC extracted the data and assessed the study quality. JPM synthesized the data, conducted the analyses, and wrote the initial draft of the manuscript, under the supervision of KC. All authors critically reviewed and revised the manuscript.

## Supporting information

Supplementary Material
