# Supplementary material for: Serious adverse drug reactions in sub‐Saharan Africa in the era of antiretroviral treatment: A systematic review
Source: Pharmacol Res Perspect. 2021 Nov 5;9(6):e00875. doi: 10.1002/prp2.875 (PMC8569857; doi:10.1002/prp2.875)

**Supplement**

**Serious adverse drug reactions in sub-Saharan Africa in the era of antiretroviral treatment: a systematic review**

JP Mouton, N Jobanputra, G Tatz, K Cohen

Division of Clinical Pharmacology, Department of Medicine, University of Cape Town

Contents

[Supplementary Data 1. List of SSA countries 3](#_Toc79395363)

[Supplementary Table S1. Search strategy for Medline via EBSCOhost 5](#_Toc79395364)

[Supplementary Table S2. Search strategy for CINAHL via EBSCOhost 6](#_Toc79395365)

[Supplementary Table S3. Search strategy for Africa-Wide Information via EBSCOhost 7](#_Toc79395366)

[Supplementary Table S4. Search strategy for Scopus 8](#_Toc79395367)

[Supplementary Table S5. Search strategy for Web of Science 9](#_Toc79395368)

[Supplementary Table S6. Search strategy for databases of theses and dissertations 10](#_Toc79395369)

[Supplementary Data 2. Data extraction form 13](#_Toc79395370)

[Supplementary Data 3. Studies excluded on full-text review, with reason for exclusion 16](#_Toc79395371)

[Supplementary Table S7. Studies reporting the proportion of admissions attributed to ADRs (Group 1 studies): heterogeneity and summary proportion, by subgroups. 18](#_Toc79395372)

[Supplementary Figure S1. Proportion of admissions attributable to ADRs (Group 1 studies), by study quality 20](#_Toc79395373)

[Supplementary Figure S2. Proportion of admissions attributable to ADRs (Group 1 studies), by study setting 21](#_Toc79395374)

[Supplementary Figure S3. Proportion of admissions attributable to ADRs (Group 1 studies), by year of data collection 22](#_Toc79395375)

[Supplementary Figure S4. Proportion of admissions attributable to ADRs (Group 1 studies), by number of study centres 23](#_Toc79395376)

[Supplementary Figure S5. Proportion of admissions attributable to ADRs (Group 1 studies), by inclusion of intensive care units 24](#_Toc79395377)

[Supplementary Figure S6. Proportion of admissions attributable to ADRs (Group 1 studies), by age groups (as reported) 25](#_Toc79395378)

[Supplementary Figure S7. Proportion of admissions attributable to ADRs (Group 1 studies), by age groups (extracting adult and paediatric datasets from Russom 2017) 26](#_Toc79395379)

[Supplementary Figure S8. Proportion of admissions attributable to ADRs (Group 1 studies), by study duration 27](#_Toc79395380)

[Supplementary Figure S9. Proportion of admissions attributable to ADRs (Group 1 studies), by ADR definition 28](#_Toc79395381)

[Supplementary Figure S10. Proportion of admissions attributable to ADRs (Group 1 studies), by ADR detection method 29](#_Toc79395382)

[Supplementary Figure S11. Proportion of admissions attributable to ADRs (Group 1 studies), by study orientation 30](#_Toc79395383)

[Supplementary Figure S12. Proportion of admissions attributable to ADRs (Group 1 studies), by surveillance team 31](#_Toc79395384)

[Supplementary Figure S13. Proportion of admissions attributable to ADRs (Group 1 studies), by folder review method 32](#_Toc79395385)

[Supplementary Figure S14. Proportion of admissions attributable to ADRs (Group 1 studies), by causality assessment method 33](#_Toc79395386)

[Supplementary Figure S15. Proportion of admissions attributable to ADRs (Group 1 studies), by causality categories included 34](#_Toc79395387)

[Supplementary Figure S16. Proportion of admissions prolonged by ADRs (Group 2 studies) 35](#_Toc79395388)

[Supplementary Figure S17. Proportion of in-hospital deaths attributable to ADRs (Group 3 studies) 36](#_Toc79395389)

### Supplementary Data 1. List of SSA countries

- Angola
- Benin
- Botswana
- Burkina Faso
- Burundi
- Cabo Verde
- Cameroon
- Central African Republic
- Chad
- Comoros
- Democratic Republic of Congo
- Republic of Congo
- Côte d’Ivoire
- Equatorial Guinea
- Eritrea
- Eswatini (Swaziland)
- Ethiopia
- Gabon
- The Gambia
- Ghana
- Guinea
- Guinea-Bissau
- Kenya
- Lesotho
- Liberia
- Madagascar
- Malawi
- Mali
- Mauritania
- Mauritius
- Mozambique
- Namibia
- Niger
- Nigeria
- Rwanda
- Sao Tome and Principe
- Senegal
- Seychelles
- Sierra Leone
- Somalia
- South Africa
- South Sudan
- Sudan
- Tanzania
- Togo
- Uganda
- Zambia
- Zimbabwe

### Supplementary Table S1. Search strategy for Medline via EBSCOhost

| **#** | **Search string** |
| --- | --- |
| S22 | LIMIT S21 to published 20020101 or later |
| S21 | S19 NOT S20 |
| S20 | PT Case Reports |
| S19 | S8 AND S11 AND S15 AND S18 |
| S18 | S16 OR S17 |
| S17 | TX (Africa OR African OR Angola OR Benin OR Botswana OR Burkina OR Volta OR Burundi OR Urundi OR Cameroon OR Cameroons OR Cameroun OR Verde OR Chad OR Comoros OR Comoro OR Comores OR Mayotte OR Congo OR Zaire OR Ivoire OR Ivory OR Eritrea OR Ethiopia OR Gabon OR Gabonese OR Gambia OR Ghana OR Guinea OR Kenya OR Lesotho OR Basutoland OR Liberia OR Madagascar OR Malagasy OR Malawi OR Nyasaland OR Mali OR Mauritania OR Mauritius OR Mozambique OR Namibia OR Niger OR Nigeria OR Rwanda OR Ruanda OR Principe OR Senegal OR Seychelles OR Sierra OR Somalia OR Sudan OR eSwatini OR Swaziland OR Tanzania OR Togo OR Uganda OR Zambia OR Zimbabwe OR Rhodesia) |
| S16 | MW ("Africa" OR "Africa South of the Sahara" OR "Africa, Central" OR "Africa, Eastern" OR "Africa, Southern" OR "Africa, Western" OR "Angola" OR "Benin" OR "Botswana" OR "Burkina Faso" OR "Burundi" OR "Cameroon" OR "Cape Verde" OR “Cabo Verde” OR "Central African Republic" OR "Chad" OR "Comoros" OR "Congo" OR "Cote d'Ivoire" OR “Ivory Coast” OR "Democratic Republic of the Congo" OR “Equatorial Guinea” OR "Eritrea" OR "Ethiopia" OR "Gabon" OR "Gambia" OR "Ghana" OR "Guinea" OR "Guinea-Bissau" OR "Kenya" OR "Lesotho" OR "Liberia" OR "Madagascar" OR "Malawi" OR "Mali" OR "Mauritania" OR "Mauritius" OR "Mozambique" OR "Namibia" OR "Niger" OR "Nigeria" OR "Rwanda" OR “Sao Tome and Principe” OR "Senegal" OR "Seychelles" OR "Sierra Leone" OR "Somalia" OR "South Africa" OR “South Sudan” OR "Sudan" OR "Swaziland" OR “eSwatini” OR "Tanzania" OR "Togo" OR "Uganda" OR "Zambia" OR "Zimbabwe") |
| S15 | S12 OR S13 OR S14 |
| S14 | TX (incidence OR prevalence OR occur* OR proportion OR rate OR number OR percent OR frequency OR epidemiology OR pharmacoepidemiology OR observational OR cohort OR prospective OR retrospective OR cross sectional OR monitor* OR surveillance) |
| S13 | MW epidemiology |
| S12 | MH ("morbidity+" OR "epidemiology+" OR "health care surveys+" OR "cohort studies+" OR "cross sectional studies+" OR "epidemiological monitoring+" OR "population surveillance+" OR "epidemiologic studies+") |
| S11 | S9 OR S10 |
| S10 | TX (serious OR seriousness OR severe OR severity OR admission OR admitt* OR hospitaliz* OR hospitalis*) |
| S9 | MH ("patient acuity+" OR "hospitalization+" OR "severity of illness index+") |
| S8 | S5 OR S6 OR S7 |
| S7 | MH ("product surveillance, postmarketing+" OR "drug-related side effects and adverse reactions+" OR "pharmacoepidemiology+" OR "iatrogenic disease") |
| S6 | TI ("ADR" OR "ADRs") |
| S5 | S1 AND S4 |
| S4 | S2 OR S3 |
| S3 | TI (adverse N3 (reaction* OR event* OR effect* OR outcome*)) |
| S2 | TI (harm* OR safety OR toxicit* OR tolerability OR problem* OR "side effect" OR iatrogenic) |
| S1 | TI (drug* OR medicine* OR medication* OR pharmaceutical* OR pharmacotherapy OR pharmacologic*) |

**TI: title. TX: full-text (title, abstract, keywords, and other fields). MH: MeSH subject heading (exact). MW: MeSH subject heading (contains word). PT: publication type. N3: proximity operator (terms occur within three words of one another, direction irrelevant). *: truncation symbol. +: explodes the subject heading (i.e., includes all MeSH headings nested under it.)**

### Supplementary Table S2. Search strategy for CINAHL via EBSCOhost

| **#** | **Search string** |
| --- | --- |
| S20 | LIMIT S19 to Human and published since 20020101; EXCLUDE Medline records |
| S19 | S8 AND S11 AND S15 AND S18 |
| S18 | S16 OR S17 |
| S17 | TX (Africa OR African OR Angola OR Benin OR Botswana OR Burkina OR Volta OR Burundi OR Urundi OR Cameroon OR Cameroons OR Cameroun OR Verde OR Chad OR Comoros OR Comoro OR Comores OR Mayotte OR Congo OR Zaire OR Ivoire OR Ivory OR Eritrea OR Ethiopia OR Gabon OR Gabonese OR Gambia OR Ghana OR Guinea OR Kenya OR Lesotho OR Basutoland OR Liberia OR Madagascar OR Malagasy OR Malawi OR Nyasaland OR Mali OR Mauritania OR Mauritius OR Mozambique OR Namibia OR Niger OR Nigeria OR Rwanda OR Ruanda OR Principe OR Senegal OR Seychelles OR Sierra OR Somalia OR Sudan OR eSwatini OR Swaziland OR Tanzania OR Togo OR Uganda OR Zambia OR Zimbabwe OR Rhodesia) |
| S16 | MW ("Africa" OR "Africa South of the Sahara" OR "Africa, Central" OR "Africa, Eastern" OR "Africa, Southern" OR "Africa, Western" OR "Angola" OR "Benin" OR "Botswana" OR "Burkina Faso" OR "Burundi" OR "Cameroon" OR "Cape Verde" OR “Cabo Verde” OR "Central African Republic" OR "Chad" OR "Comoros" OR "Congo" OR "Cote d'Ivoire" OR "Democratic Republic of the Congo" OR “Equatorial Guinea” OR "Eritrea" OR "Ethiopia" OR "Gabon" OR "Gambia" OR "Ghana" OR "Guinea" OR "Guinea-Bissau" OR "Kenya" OR "Lesotho" OR "Liberia" OR "Madagascar" OR "Malawi" OR "Mali" OR "Mauritania" OR "Mauritius" OR "Mozambique" OR "Namibia" OR "Niger" OR "Nigeria" OR "Rwanda" OR “Sao Tome and Principe” OR "Senegal" OR "Seychelles" OR "Sierra Leone" OR "Somalia" OR "South Africa" OR “South Sudan” OR "Sudan" OR "Swaziland" OR "Tanzania" OR "Togo" OR "Uganda" OR "Zambia" OR "Zimbabwe") |
| S15 | S12 OR S13 OR S14 |
| S14 | TX (incidence OR prevalence OR occur* OR proportion OR rate OR number OR percent OR frequency OR epidemiology OR pharmacoepidemiology OR observational OR cohort OR prospective OR retrospective OR cross sectional OR monitor* OR surveillance) |
| S13 | MW (morbidity OR mortality) |
| S12 | MH (Epidemiology+ OR “Nonexperimental Studies+") |
| S11 | S9 OR S10 |
| S10 | TX (serious OR seriousness OR severe OR severity OR admission OR admitt* OR hospitaliz* OR hospitalis* OR death OR dying OR died OR fatal* OR mortal*) |
| S9 | MH (“patient classification" OR "institutionalization+" OR "mortality+" OR "severity of illness indices+") |
| S8 | S5 OR S6 OR S7 |
| S7 | MH ("drug evaluation+" OR "adverse drug events+" OR “drug toxicity+” OR "pharmacovigilance+" OR "iatrogenic disease+" OR “drug therapy+/ae/mo”) |
| S6 | TI ("ADR" OR "ADRs") |
| S5 | S1 AND S4 |
| S4 | S2 OR S3 |
| S3 | TI (adverse N3 (reaction* OR event* OR effect* OR outcome*)) |
| S2 | TI (harm* OR safety OR toxicit* OR tolerability OR problem* OR "side effect" OR iatrogenic) |
| S1 | TI (drug* OR medicine* OR medication* OR pharmaceutical* OR pharmacotherapy OR pharmacologic*) |

**TI: title. TX: full-text (title, abstract, keywords, and other fields). MH: CINAHL subject heading (exact). MW: CINAHL subject heading (contains word). N3: proximity operator (terms occur within three words of one another, direction irrelevant). *: truncation symbol. +: explodes the subject heading (i.e., includes all CINAHL headings nested under it.) /ae: topical subheading “adverse effects”. /mo: topical subheading “mortality”**

### Supplementary Table S3. Search strategy for Africa-Wide Information via EBSCOhost

| **#** | **Search string** |
| --- | --- |
| S17 | LIMIT S16 to Publication year 2002 - |
| S16 | S8 AND S11 AND S14 AND S15 |
| S15 | TX (Africa OR African OR Angola OR Benin OR Botswana OR Burkina OR Volta OR Burundi OR Urundi OR Cameroon OR Cameroons OR Cameroun OR Verde OR Chad OR Comoros OR Comoro OR Comores OR Mayotte OR Congo OR Zaire OR Ivoire OR Ivory OR Eritrea OR Ethiopia OR Gabon OR Gabonese OR Gambia OR Ghana OR Guinea OR Kenya OR Lesotho OR Basutoland OR Liberia OR Madagascar OR Malagasy OR Malawi OR Nyasaland OR Mali OR Mauritania OR Mauritius OR Mozambique OR Namibia OR Niger OR Nigeria OR Rwanda OR Ruanda OR Principe OR Senegal OR Seychelles OR Sierra OR Somalia OR Sudan OR eSwatini OR Swaziland OR Tanzania OR Togo OR Uganda OR Zambia OR Zimbabwe OR Rhodesia) |
| S14 | S12 OR S13 |
| S13 | TX (incidence OR prevalence OR occur* OR proportion OR rate OR number OR percent OR frequency OR epidemiology OR pharmacoepidemiology OR observational OR cohort OR prospective OR retrospective OR cross sectional OR monitor* OR surveillance) |
| S12 | SU (morbidit* OR mortal* OR epidemiol* OR incidence OR prevalence OR proportion OR rates OR observational OR prospective OR retrospective OR pharmacoepidemiology OR "health care surveys” OR "cohort study" OR "cross sectional" OR "population surv*”) |
| S11 | S9 OR S10 |
| S10 | TX (serious OR seriousness OR severe OR severity OR admission OR admitt* OR hospitaliz* OR hospitalis* OR death OR dying OR died OR fatal* OR mortal*) |
| S9 | SU (hospitalization OR hospital admission OR hospital readmission OR admission OR readmission OR hospitalisation OR hospitalized OR hospitalised OR admit* OR patient acuity OR severe OR severity OR serious OR seriousness OR death OR died OR dying OR mortal* OR fatal*) |
| S8 | S5 OR S6 OR S7 |
| S7 | SU (“adverse drug” OR “side effects” OR pharmacovigilance OR pharmacoepidemiology OR “drug related” OR iatrogenic) |
| S6 | TI ("ADR" OR "ADRs") |
| S5 | S1 AND S4 |
| S4 | S2 OR S3 |
| S3 | TI (adverse N3 (reaction* OR event* OR effect* OR outcome*)) |
| S2 | TI (harm* OR safety OR toxicit* OR tolerability OR problem* OR "side effect" OR iatrogenic) |
| S1 | TI (drug* OR medicine* OR medication* OR pharmaceutical* OR pharmacotherapy OR pharmacologic*) |

**TI: title. TX: full-text (title, abstract, keywords, and other fields). SU: subject. N3: proximity operator (terms occur within three words of one another, direction irrelevant). *: truncation symbol.**

### Supplementary Table S4. Search strategy for Scopus

| ( ( ( ( TITLE ( drug* OR medicine* OR medication* OR pharmaceutical* OR pharmacotherapy OR pharmacologic* ) ) AND ( ( TITLE ( harm* OR safety OR toxicit* OR tolerability OR problem* OR "side effect" OR iatrogenic ) ) OR ( TITLE ( adverse W/3 ( reaction* OR event* OR effect* OR outcome* ) ) ) ) ) OR ( TITLE ( "ADR" OR "ADRs" ) ) OR ( INDEXTERMS ( "adverse drug reaction" OR "drug induced disease" OR "pharmacovigilance" OR "drug fatality" OR "drug safety" OR "drug surveillance program" OR "pharmacoepidemiology" OR "unspecified side effect" ) ) ) ) AND ( ( INDEXTERMS ( "hospital admission" OR "hospitalization" OR "hospital patient" OR "mortality" ) ) OR ( TITLE-ABS-KEY ( serious OR seriousness OR severe OR severity OR admission OR admitt* OR hospitaliz* OR hospitalis* OR death OR dying OR died OR fatal* OR mortal* ) ) ) AND ( ( INDEXTERMS ( "morbidity" OR "mortality" OR "cross-sectional study" OR "health survey" OR "medical record review" OR "population research" OR "incidence" OR "prevalence" OR "retrospective study" OR "prospective study" OR "cohort analysis" OR "longitudinal study" OR "controlled study" ) ) OR ( TITLE-ABS-KEY ( incidence OR prevalence OR occur* OR proportion OR rate OR number OR percent OR frequency OR epidemiology OR pharmacoepidemiology OR observational OR cohort OR prospective OR retrospective OR cross AND sectional OR monitor* OR surveillance ) ) ) AND ( ( ( INDEXTERMS ( "Africa" OR "Africa South of the Sahara" OR "Africa, Central" OR "Africa, Eastern" OR "Africa, Southern" OR "Africa, Western" OR "Angola" OR "Benin" OR "Botswana" OR "Burkina Faso" OR "Burundi" OR "Cameroon" OR "Cape Verde" OR "Central African Republic" OR "Chad" OR "Comoros" OR "Congo" OR "Cote d'Ivoire" OR “Ivory Coast” OR "Democratic Republic of the Congo" OR "Equatorial Guinea" OR "Eritrea" OR "Ethiopia" OR "Gabon" OR "Gambia" OR "Ghana" OR "Guinea" OR "Guinea-Bissau" OR "Kenya" OR "Lesotho" OR "Liberia" OR "Madagascar" OR "Malawi" OR "Mali" OR "Mauritania" OR "Mauritius" OR "Mozambique" OR "Namibia" OR "Niger" OR "Nigeria" OR "Rwanda" OR "Sao Tome and Principe" OR "Senegal" OR "Seychelles" OR "Sierra Leone" OR "Somalia" OR "South Africa" OR "South Sudan" OR "Sudan" OR "Swaziland" OR “eSwatini” OR "Tanzania" OR "Togo" OR "Uganda" OR "Zambia" OR "Zimbabwe" ) ) ) OR ( TITLE-ABS-KEY ( africa OR angola OR benin OR botswana OR burkina OR volta OR burundi OR urundi OR cameroon OR cameroons OR cameroun OR verde OR chad OR comoros OR comoro OR comores OR mayotte OR congo OR zaire OR ivoire OR ivory OR eritrea OR ethiopia OR gabon OR gabonese OR gambia OR ghana OR guinea OR kenya OR lesotho OR basutoland OR liberia OR madagascar OR malagasy OR malawi OR nyasaland OR mali OR mauritania OR mauritius OR mozambique OR namibia OR niger OR nigeria OR rwanda OR ruanda OR principe OR senegal OR seychelles OR sierra OR somalia OR sudan OR eswatini OR swaziland OR tanzania OR togo OR uganda OR zambia OR zimbabwe OR rhodesia ) ) ) AND (LIMIT-TO ( PUBYEAR , 2021 ) OR LIMIT-TO ( PUBYEAR , 2020 ) OR LIMIT-TO ( PUBYEAR , 2019 ) OR LIMIT-TO ( PUBYEAR , 2018 ) OR LIMIT-TO ( PUBYEAR , 2017 ) OR LIMIT-TO ( PUBYEAR , 2016 ) OR LIMIT-TO ( PUBYEAR , 2015 ) OR LIMIT-TO ( PUBYEAR , 2014 ) OR LIMIT-TO ( PUBYEAR , 2013 ) OR LIMIT-TO ( PUBYEAR , 2012 ) OR LIMIT-TO ( PUBYEAR , 2011 ) OR LIMIT-TO ( PUBYEAR , 2010 ) OR LIMIT-TO ( PUBYEAR , 2009 ) OR LIMIT-TO ( PUBYEAR , 2008 ) OR LIMIT-TO ( PUBYEAR , 2007 ) OR LIMIT-TO ( PUBYEAR , 2006 ) OR LIMIT-TO ( PUBYEAR , 2005 ) OR LIMIT-TO ( PUBYEAR , 2004 ) OR LIMIT-TO ( PUBYEAR , 2003 ) OR LIMIT-TO ( PUBYEAR , 2002 ) ) AND ( LIMIT-TO ( DOCTYPE , "ar" ) OR LIMIT-TO ( DOCTYPE , "no" ) OR LIMIT-TO ( DOCTYPE , "le" ) OR LIMIT-TO ( DOCTYPE , "cp" ) OR LIMIT-TO ( DOCTYPE , "sh" ) OR LIMIT-TO ( DOCTYPE , "ch" ) OR LIMIT-TO ( DOCTYPE , "cr" ) ) AND ( EXCLUDE ( EXACTKEYWORD , "Nonhuman" ) OR EXCLUDE ( EXACTKEYWORD , "Animal Experiment" ) OR EXCLUDE ( EXACTKEYWORD , "Animal Model" ) ) AND NOT INDEX(medline) |
| --- |

**TITLE-ABS-KEY: title, abstract, keywords. W/3: proximity operator (terms occur within three words of one another, direction irrelevant). *: truncation symbol.**

### Supplementary Table S5. Search strategy for Web of Science

| **#** | **Search string** |
| --- | --- |
| #14 | Refine #13 by excluding MEDLINE |
| #13 | LIMIT #12 to Publication years 2002- |
| #12 | #11 AND #10 AND #9 AND #8 |
| #11 | TS=(Africa OR Angola OR Benin OR Botswana OR Burkina OR Volta OR Burundi OR Urundi OR Cameroon OR Cameroons OR Cameroun OR Verde OR Chad OR Comoros OR Comoro OR Comores OR Mayotte OR Congo OR Zaire OR Ivoire OR Ivory OR Eritrea OR Ethiopia OR Gabon OR Gabonese OR Gambia OR Ghana OR Guinea OR Kenya OR Lesotho OR Basutoland OR Liberia OR Madagascar OR Malagasy OR Malawi OR Nyasaland OR Mali OR Mauritania OR Mauritius OR Mozambique OR Namibia OR Niger OR Nigeria OR Rwanda OR Ruanda OR Principe OR Senegal OR Seychelles OR Sierra OR Somalia OR Sudan OR eSwatini OR Swaziland OR Tanzania OR Togo OR Uganda OR Zambia OR Zimbabwe OR Rhodesia) |
| #10 | TS=(morbidity OR mortality OR epidemiology OR survey OR cohort OR "cross sectional" OR incidence OR prevalence OR proportion OR pharmacoepidemiology OR observational OR prospective OR retrospective OR monitoring) |
| #9 | TS=(hospitalization OR admission OR mortality OR death OR fatal OR severe OR serious) |
| #8 | #5 OR #6 OR #7 |
| #7 | TS=(“postmarketing product surveillance" OR "adverse drug reaction*" OR “adverse drug event*” OR pharmacoepidemiology OR "iatrogenic disease") |
| #6 | TI=("ADR" OR "ADRs") |
| #5 | #1 AND #4 |
| #4 | #2 OR #3 |
| #3 | TI=(adverse NEAR/3 (reaction OR event OR effect OR outcome)) |
| #2 | TI=(harm OR safety OR toxicity OR tolerability OR problem OR "side effect*" OR iatrogenic) |
| #1 | TI=(drug OR medicine OR medication OR pharmaceutical OR pharmacotherapy OR pharmacologic*) |

**TI: title search. TS: topic search *: truncation symbol.**

### Supplementary Table S6. Search strategy for databases of theses and dissertations

| **Database** | **Interface** | **Search string** |
| --- | --- | --- |
| Networked Digital Library of Theses and Dissertations (NDLTD) | search.ndltd.org | **(subject:"pharmacovigilance" OR subject:"pharmacoepidemiology" OR title:"adverse drug") AND (subject:"Africa" OR subject:"Sub-Saharan Africa" OR subject:"Angola" OR subject:"Benin" OR subject:"Botswana" OR subject:"Burkina Faso" OR subject:"Burundi" OR subject:"Cabo Verde" OR subject:"Cameroon" OR subject:"Central African Republic" OR subject:"Chad" OR subject:"Comoros" OR subject:"Democratic Republic of Congo" OR subject:"Republic of Congo" OR subject:"Cote d'Ivoire" OR subject:"Equatorial Guinea" OR subject:"Eritrea" OR subject:"Ethiopia" OR subject:"Gabon" OR subject:"Gambia" OR subject:"Ghana" OR subject:"Guinea" OR subject:"Guinea-Bissau" OR subject:"Kenya" OR subject:"Lesotho" OR subject:"Liberia" OR subject:"Madagascar" OR subject:"Malawi" OR subject:"Mali" OR subject:"Mauritania" OR subject:"Mauritius" OR subject:"Mozambique" OR subject:"Namibia" OR subject:"Niger" OR subject:"Nigeria" OR subject:"Rwanda" OR subject:"Sao Tome and Principe" OR subject:"Senegal" OR subject:"Seychelles" OR subject:"Sierra Leone" OR subject:"Somalia" OR subject:"South Africa" OR subject:"South Sudan" OR subject:"Sudan" OR subject:"Swaziland" OR subject:"Tanzania" OR subject:"Togo" OR subject:"Uganda" OR subject:"Zambia" OR subject:"Zimbabwe")**  Restrict to publication years 2002 to 2021 |
| WorldCatDissertations | OCLC firstsearch via UCT libraries | **((ti: adverse n3 drug) OR (kw: adverse n3 drug) OR kw: pharmacoepidemiology OR kw: pharmacovigilance) AND (kw: Africa OR kw: Sub-Saharan Africa OR kw: Angola OR kw: Benin OR kw: Botswana OR kw: Burkina Faso OR kw: Burundi OR kw: Cabo Verde OR kw: Cameroon OR kw: Central African Republic OR kw: Chad OR kw: Comoros OR kw: Democratic Republic of Congo OR kw: Republic of Congo OR kw: Cote d'Ivoire OR kw: Equatorial Guinea OR kw: Eritrea OR kw: Ethiopia OR kw: Gabon OR kw: Gambia OR kw: Ghana OR kw: Guinea OR kw: Guinea-Bissau OR kw: Kenya OR kw: Lesotho OR kw: Liberia OR kw: Madagascar OR kw: Malawi OR kw: Mali OR kw: Mauritania OR kw: Mauritius OR kw: Mozambique OR kw: Namibia OR kw: Niger OR kw: Nigeria OR kw: Rwanda OR kw: Sao Tome and Principe OR kw: Senegal OR kw: Seychelles OR kw: Sierra Leone OR kw: Somalia OR kw: South Africa OR kw: South Sudan OR kw: Sudan OR kw: Swaziland OR kw: Tanzania OR kw: Togo OR kw: Uganda OR kw: Zambia OR kw: Zimbabwe) AND yr: 2002-2021** |
| ProQuest Dissertations & Theses A&I | UCT libraries | **(diskw.Exact("Adverse drug event" OR "adverse side reactions" OR "Adverse side-effects" OR "Side effect" OR "Iatrogenic effects" OR "Adverse events in patients" OR "Hospital-acquired adverse events" OR "Adverse outcome" OR "Adverse drug events" OR "Adverse drug reaction(adr)" OR "Adverse drug safety" OR "Drug Safety" OR "Side effects (treatment)" OR "Metabolic adverse events" OR "Iatrogenic" OR "Iatrogenic harm" OR "Drug side effect" OR "Adverse outcomes" OR "Iatrogenic disease" OR "Drug related problems" OR "Adverse drug reactions" OR "Side-effect" OR "Rare adverse drug effects" OR "Adverse drug event detection" OR "Drug-related deaths" OR "Drug-related death" OR "Preventable adverse events" OR "Adverse Effects" OR "Adverse event reaction" OR "ADR" OR "Adverse side effects" OR "Severe adverse event" OR "Adverse effect" OR "Adr reporting systems" OR "Adverse event" OR "Adverse Drug Reaction" OR "Drugs side effect" OR "Adverse Event Reporting System" OR "Side-effect effect" OR "Adverse events" OR "Side effects") OR ALL(pharmacoepidemiology OR pharmacovigilance OR "adverse drug")) AND LOC("Africa" OR "Sub-Saharan Africa" OR "Angola" OR "Benin" OR "Botswana" OR "Burkina Faso" OR "Burundi" OR "Cabo Verde" OR "Cameroon" OR "Central African Republic" OR "Chad" OR "Comoros" OR "Democratic Republic of Congo" OR "Republic of Congo" OR "Cote d'Ivoire" OR "Equatorial Guinea" OR "Eritrea" OR "Ethiopia" OR "Gabon" OR "Gambia" OR "Ghana" OR "Guinea" OR "Guinea-Bissau" OR "Kenya" OR "Lesotho" OR "Liberia" OR "Madagascar" OR "Malawi" OR "Mali" OR "Mauritania" OR "Mauritius" OR "Mozambique" OR "Namibia" OR "Niger" OR "Nigeria" OR "Rwanda" OR "Sao Tome and Principe" OR "Senegal" OR "Seychelles" OR "Sierra Leone" OR "Somalia" OR "South Africa" OR "South Sudan" OR "Sudan" OR "Swaziland" OR "Tanzania" OR "Togo" OR "Uganda" OR "Zambia" OR "Zimbabwe") AND (PD(2002) OR PD(2003) OR PD(2004) OR PD(2005) OR PD(2006) OR PD(2007) OR PD(2008) OR PD(2009) PD(2010) OR PD(2011) OR PD(2012) OR PD(2013) OR PD(2014) OR PD(2015) OR PD(2016) OR PD(2017) OR PD(2018) OR PD(2019) OR PD(2020) OR PD(2021))** |
| Openthesis | [www.openthesis.org](http://www.openthesis.org) | ((**TI("adverse") AND TI("drug")) OR (IF("adverse") AND IF("drug")) OR IF("pharmacoepidemiology") OR IF("pharmacovigilance")) AND (IF("Africa") OR IF("Sub-Saharan Africa") OR IF("Angola") OR IF("Benin") OR IF("Botswana") OR IF("Burkina Faso") OR IF("Burundi") OR IF("Cabo Verde") OR IF("Cameroon") OR IF("Central African Republic") OR IF("Chad") OR IF("Comoros") OR IF("Democratic Republic of Congo") OR IF("Republic of Congo") OR IF("Cote d'Ivoire") OR IF("Equatorial Guinea") OR IF("Eritrea") OR IF("Ethiopia") OR IF("Gabon") OR IF("Gambia") OR IF("Ghana") OR IF("Guinea") OR IF("Guinea-Bissau") OR IF("Kenya") OR IF("Lesotho") OR IF("Liberia") OR IF("Madagascar") OR IF("Malawi") OR IF("Mali") OR IF("Mauritania") OR IF("Mauritius") OR IF("Mozambique") OR IF("Namibia") OR IF("Niger") OR IF("Nigeria") OR IF("Rwanda") OR IF("Sao Tome and Principe") OR IF("Senegal") OR IF("Seychelles") OR IF("Sierra Leone") OR IF("Somalia") OR IF("South Africa") OR IF("South Sudan") OR IF("Sudan") OR IF("Swaziland") OR IF("Tanzania") OR IF("Togo") OR IF("Uganda") OR IF("Zambia") OR IF("Zimbabwe"))** |
| Open Access Theses and Dissertations | oatd.org | **title:((adverse AND drug) OR subject:(adverse AND drug) OR subject:(pharmacoepidemiology OR pharmacovigilance)) AND (subject: (Africa OR "Sub-Saharan Africa" OR Angola OR Benin OR Botswana OR "Burkina Faso" OR Burundi OR "Cabo Verde" OR Cameroon OR "Central African Republic" OR Chad OR Comoros OR "Democratic Republic of Congo" OR "Republic of Congo" OR "Cote d'Ivoire" OR "Equatorial Guinea" OR Eritrea OR Ethiopia OR Gabon OR Gambia OR Ghana OR Guinea OR Guinea-Bissau OR Kenya OR Lesotho OR Liberia OR Madagascar OR Malawi OR Mali OR Mauritania OR Mauritius OR Mozambique OR Namibia OR Niger OR Nigeria OR Rwanda OR "Sao Tome and Principe" OR Senegal OR Seychelles OR "Sierra Leone" OR Somalia OR "South Africa" OR "South Sudan" OR Sudan OR Swaziland OR Tanzania OR Togo OR Uganda OR Zambia OR Zimbabwe))** |

### Supplementary Data 2. Data extraction form

| 1. ***Study ID and inclusion criteria:***     1. **First author surname:** _________    2. **Year of publication:** _________    3. **Completeness:** Abstract only \| Full-text    4. **Does this study contain:**        1. Data on admissions due to ADRs: Yes \| No – if yes, complete sections 2, 3, and 4.       2. Data on prolongation of hospitalisation due to ADRs: Yes \| No – if yes, complete sections 2, 3, and 5.       3. Data on in-hospital deaths due to ADRs: Yes \| No – if yes, complete section 2, 3, and 6. 2. ***Survey setting and population (complete in all cases):***    1. **Country** (if not stated, then corresponding author address determines): ­_________    2. **Year of data collection:** ________ \| not stated    3. **Number of hospitals:** Single-centre \| multicentre    4. **Hospital(s) surveyed:** Primary only (= "community") \| Secondary / tertiary (=  "teaching" / "university" / "specialist" / "referral" / "regional") / mixture \| not stated    5. **Wards surveyed:**        1. Medical wards n.o.s.: Yes \| No \| not stated       2. Medical speciality wards: No \| not stated \| Yes, specify: _______________ (e.g. cardiology / nephrology / neurology)       3. ICU: Yes \| No \| not stated       4. Oncology: Yes \| No \| not stated       5. Surgical wards: Yes \| No \| not stated       6. Paediatric wards: Yes \| No \| not stated       7. Geriatric wards: Yes \| No \| not stated    6. **Patients surveyed:**       1. All pts \| Random sample of pts \| Non-randomised sample (describe) ________________       2. Did original authors have any specific exclusions in their survey? If yes, describe: ________________       3. Proportion of potentially identified patients excluded from the survey: ________    7. **Duration of survey:** _________ \| not stated 3. ***Study methodology (complete in all cases):***    1. ***ADE* identification:** Spontaneous reporting \| Stimulated spontaneous reporting \| Automated (computerised) EMR / labs surveillance \| Active (human) surveillance \| not stated or unclear    2. If **Active surveillance:**       1. **Surveillance team:** single investigator \| unidisciplinary team \| multidisciplinary team \| not stated or unclear       2. **Surveillance source material:** folder review only (including review of lab results) \| folder review and additional methods (e.g. spontaneous reports / pt interview etc.) \| not stated or unclear       3. **Surveillance orientation:** Prospective \| Retrospective \| not stated or unclear    3. ***ADR* determination:**        1. Was an **ADR determination (= causality assessment) done**? Yes \| No \| not stated or unclear       2. **ADR definition used:** WHO \| Aronson \| not stated \| other: __________       3. Regardless of claimed definition, do the authors include any of the following as an ADR?          1. Adverse reactions to herbal / traditional medicines: Yes \| No \| unclear or not stated          2. accidental overdose: Yes \| No \| unclear or not stated          3. intentional overdose: Yes \| No \| unclear or not stated          4. non-compliance: Yes \| No \| unclear or not stated          5. drug abuse: Yes \| No \| unclear or not stated          6. medication error: Yes \| No \| unclear or not stated          7. therapeutic failure: Yes \| No \| unclear or not stated       4. What **evidence available to make ADR determination on?** Spontaneous report ("Yellow card") \| Summary of case notes (incl lab results) \| Full case notes (= that which was available to clinical team) \| Full case notes and additionally sourced evidence not available to clinical team, such as primary care records \| not stated or unclear       5. **Causality assessment:**          1. Causality assessment **by whom?** single investigator \| unidisciplinary team \| multidisciplinary team \| not stated or unclear          2. Causality assessment done by **same team as ADE identification team?** Yes \| No \| not stated or unclear          3. Which **tool**? WHO-UMC \| Naranjo \| not stated or unclear \| other_________          4. **Which causality levels were included as ADRs?** Only definite and probable \| Definite, probable, and possible \| Other ________    4. **Seriousness determination:**       1. **Done?** Yes, formal seriousness determination done \| No, seriousness implied as study only reports on ADE/ADR *causing* admission/prolongation/death       2. Seriousness assessment **by whom?** single investigator \| unidisciplinary team \| multidisciplinary team \| not stated or unclear       3. Seriousness assessment done by **same team** as ADE identification team? Yes \| No \| not stated or unclear       4. Which **tool**? Hartwig \| not stated or unclear \| other _________    5. **Preventability determination:**       1. **Done?** Yes \| No       2. Preventability assessment **by whom?** single investigator \| unidisciplinary team \| multidisciplinary team \| not stated or unclear       3. Preventability assessment done by **same team** as ADE identification team? Yes \| No \| not stated or unclear       4. Which **tool**? Schumock \| Hallas \| not stated or unclear \| other _________ 4. ***ADR-RELATED ADMISSIONS – Survey findings:***    1. **Denominator:**       1. No. of **patients** ____ and/or No. of **admissions** ___ and/or No. of **patients on drugs** _____ and/or No. of **admissions on drugs** ____       2. **Age** of patients in the denominator: mean ____ and/or median ___ and/or IQR ___ and/or range ___       3. Proportion of denominator **female**: ____       4. Proportion of denominator **HIV-infected:** ______       5. **Other characteristics** of the patients in the denominator (notable morbidity, specific exclusions, etc.): _______________    2. **Numerator:**       1. No of **patients with ADR-related admission** ___ and/or **No. of admissions that are ADR-related** ____ (note this is not ADRs present on admission, but ADRs implied as reason for admission)       2. No of **patients with preventable ADR-related admission**: ___and/or **No. of admissions that are related to preventable ADRs:**______ \| not stated       3. Does the study mention the **most common clinical presentations** of ADRs resulting in admission? Yes \| No. If yes, list up to top-5, with frequencies: ____________       4. Does the study mention the **most common drugs** implicated in ADR-related admission? Yes \| No. If yes, list up to top-5, with frequencies: ___________       5. Does the study mention the **most common drug classes** implicated in ADR-related admissions? Yes \| No. If yes, list up to top-5, with frequencies: ____________       6. **Other notable characteristics** of the patients in the numerator, if mentioned (for example, HIV status): ______ 5. ***ADR-RELATED PROLONGATION OF HOSPITAL STAY – Survey findings:***    1. **Denominator:**       1. No. of **patients** ____ and/or No. of **admissions** ___ and/or No. of **patients on drugs** _____ and/or No. of **admissions on drugs** ____       2. **Age** of patients in the denominator: mean ___ and/or median ___ and/or IQR ___ and/or range ___       3. Proportion of denominator **female**: ____       4. **Other characteristics** of patients in the denominator (notable morbidity, specific exclusions, etc.): _______________    2. **Numerator:**       1. No of **patients with ADR-related prolongation of hospital stay** ___ and/or **No. of admissions prolonged by ADR** ____       2. No of **patients with preventable ADR-related prolongation of stay**: ___and/or **No. of admissions prolonged by ADRs that were preventable:**______ \| not stated       3. Does the study mention the **most common clinical presentations** of ADRs resulting in prolongation of stay? Yes \| No. If yes, list up to top-5, with frequencies: ____________       4. Does the study mention the **most common drugs** implicated in ADR-related prolongation of stay? Yes \| No. If yes, list up to top-5, with frequencies: ___________       5. Does the study mention the **most common drug classes** implicated in ADR-related admissions? Yes \| No. If yes, list up to top-5, with frequencies: ____________       6. **Other notable characteristics** of the patients in the numerator, if mentioned (for example, HIV status): ______ 6. ***ADR-RELATED DEATHS – Survey findings:***    1. **Denominator:** No of in-hospital deaths: __________    2. **Numerator:** No of in-hospital deaths that were ADR-related: ____________    3. **Numerator:** No of in-hospital deaths that were attributed to **preventable** ADRs: ___________    4. Does the study mention the **most common clinical presentations** of ADRs resulting in in-hospital death? Yes \| No. If yes, list up to top-5, with frequencies: ____________    5. Does the study mention the **most common drugs** implicated in ADR-related in-hospital death? Yes \| No. If yes, list up to top-5, with frequencies: ___________    6. Does the study mention the **most common drug classes** implicated in ADR-related admissions? Yes \| No. If yes, list up to top-5, with frequencies: ____________    7. **Other notable characteristics** of the patients in the numerator, if mentioned (for example, HIV status): ______ |
| --- |

### Supplementary Data 3. Studies excluded on full-text review, with reason for exclusion

***Not in sub-Saharan Africa***

1. Iyer, K., et al., *Comparison of Drug Related Problems Associated with Use of Narrow Therapeutic Index Drugs and Other Drugs in Hospitalized Patients.* Journal of Young Pharmacists, 2018. **10**(3):318-321.
2. McClead, R.E., Jr., et al., *An internal quality improvement collaborative significantly reduces hospital-wide medication error related adverse drug events.* J Pediatr, 2014. **165**(6):1222-1229.

***Not primary research***

1. Blockman, M., *Adverse drug reactions - an update.* CME, 2009. **27**(2):80-83.
2. Mekonnen, A.B., et al., *Adverse Drug Events and Medication Errors in African Hospitals: A Systematic Review.* Drugs Real World Outcomes, 2018. **5**(1):1-24.

***Not in a hospital***

1. Elhadji, B.A.K., et al., *A surveillance system to measure childhood mortality and drug related adverse events in three districts in Senegal*, in *American Society of Tropical Medicine and Hygiene 59th Annual Meeting*. 2010, American Journal of Tropical Medicine and Hygiene: Atlanta. p. 361.

***Not in unselected patients***

1. Abrogoua, D.P., et al., *Pharmaceutical interventions in the management of tuberculosis in a pneumophtisiology department, Ivory Coast.* Ther Clin Risk Manag, 2016. **12**:1749-1756.
2. Bekele, F., et al., *Drug-related problems among patients with infectious disease admitted to medical wards of Wollega University Referral Hospital: Prospective observational study.* SAGE Open Med, 2021. **9**:2050312121989625.

***Emergency unit presentations***

1. Kamagaté M., Die-Kacou H., Yavo J.-C., et al*.* *Impact pharmacoeconomic of avoidable adverse drug reactions in emergencies*. Abstract presented at Congrès de Physiologie, de Pharmacologie, et de Thérapeutique, 23 to 25 March 2010, Bordeaux. Fundam Clin Pharmacol, 2010. **24**:92.
2. Mouton, J.P., et al., *Adult medical emergency unit presentations due to adverse drug reactions in a setting of high HIV prevalence.* Afr J Emerg Med, 2021. **11**(1):46-52.
3. Tipping, B., et al., *The burden and risk factors for adverse drug events in older patients - a prospective cross-sectional study*. South African Medical Journal, 2006. **96**(12): 1255-1259.

***No disaggregated numerator***

1. Ahmed, S.M., et al., *Medication-related problems among hospitalized pregnant women in a tertiary teaching hospital in Ethiopia: a prospective observational study.* BMC Pregnancy Childbirth, 2020. **20**(1):737.
2. Belayneh, Y.M., G. Amberbir, and A. Agalu, *A prospective observational study of drug therapy problems in medical ward of a referral hospital in northeast Ethiopia.* BMC Health Serv Res, 2018. **18**(1):808.
3. Birarra, M.K., T.B. Heye, and W. Shibeshi, *Assessment of drug-related problems in pediatric ward of Zewditu Memorial Referral Hospital, Addis Ababa, Ethiopia.* Int J Clin Pharm, 2017. **39**(5):1039-1046.
4. Bizuneh, G.K., et al., *A Prospective Observational Study of Drug Therapy Problems in Pediatric Ward of a Referral Hospital, Northeastern Ethiopia.* Int J Pediatr, 2020. **2020**:4323189.
5. Dedefo, M.G., A.H. Mitike, and M.T. Angamo, *Incidence and determinants of medication errors and adverse drug events among hospitalized children in West Ethiopia.* BMC Pediatr, 2016. **16**:81.
6. Eshetie, T.C., et al., *Adverse drug events in hospitalized children at Ethiopian University Hospital: a prospective observational study.* BMC Pediatr, 2015. **15**:83.
7. Eze, U.I.H., C.A. Oparah, and F.A. Lawal, *Identification of Drug Therapy Problems among Elderly in-patients of a Nigerian Teaching Hospital.* Nigerian Journal of Pharmaceutical Research, 2011. **9**(1):49-56.
8. Hailu, B.Y., et al., *Drug related problems in admitted geriatric patients: the impact of clinical pharmacist interventions.* BMC Geriatr, 2020. **20**(1):13.
9. Gokhul, A., P.M. Jeena, and A. Gray, *Iatrogenic medication errors in a paediatric intensive care unit in Durban, South Africa.* S Afr Med J, 2016. **106**(12):1222-1229.
10. Kiguba, R., C. Karamagi, and S.M. Bird, *Incidence, risk factors and risk prediction of hospital-acquired suspected adverse drug reactions: a prospective cohort of Ugandan inpatients.* BMJ Open, 2017. **7**(1):e010568.
11. Matsaseng, T. and J. Moodley, *Adverse events in gynaecology at King Edward VIII Hospital, Durban, South Africa.* J Obstet Gynaecol, 2005. **25**(7):676-80.
12. Müller, M., A. Gous, and N. Schellack, *Measuring adverse events using a trigger tool in a paper based patient information system at a teaching hospital in South Africa.* European Journal of Clinical Pharmacy, 2016. **18**(2):103-110.
13. Sahilu, T., et al., *Adverse Drug Events and Contributing Factors Among Hospitalized Adult Patients at Jimma Medical Center, Southwest Ethiopia: A Prospective Observational Study.* Curr Ther Res Clin Exp, 2020. **93**:100611.
14. Saka, S.A., M. Nlooto, and F. Oosthuizen, *American Geriatrics Society-Beers Criteria and adverse drug reactions: a comparative cross-sectional study of Nigerian and South African older inpatients.* Clin Interv Aging, 2018. **13**:2375-2387.
15. Tigabu, B.M., D. Daba, and B. Habte, *Drug-related problems among medical ward patients in Jimma university specialized hospital, Southwest Ethiopia.* J Res Pharm Pract, 2014. **3**(1):1-5.
16. Zeleke, A., T. Chanie, and M. Woldie, *Medication prescribing errors and associated factors at the pediatric wards of Dessie Referral Hospital, Northeast Ethiopia.* Int Arch Med, 2014. **7**:18.

### Supplementary Table S7. Studies reporting the proportion of admissions attributed to ADRs (Group 1 studies): heterogeneity and summary proportion, by subgroups.

|  | **Number of studies** | **I^2^** | **Proportion of admissions attributed to ADRs (median [IQR])** |
| --- | --- | --- | --- |
| Overall | 14 | 98.2% | 4.8% [1.5% to 7.0%] |
| Study quality |  |  |  |
| Lowest tercile | 5 | 95.7% | 2.6% [1.5% to 6.4%] |
| Mid tercile | 2 | - | 6.3% [5.7% to 7.0%] |
| Highest tercile | 7 | 97.9% | 4.0% [1.5% to 8.4%] |
| Setting |  |  |  |
| Nigeria | 5 | 97.0% | 1.5% [0.6% to 4.0%] |
| South Africa | 4 | 95.5% | 5.9% [3.7% to 7.3%] |
| Other | 5 | 95.4% | 7.0% [2.6% to 7.1%] |
| Year of data collection |  |  |  |
| 2006 or earlier | 4 | 96.5% | 2.0% [1.0% to 4.4%] |
| 2007 to 2012 | 3 | - | 6.4% [0.6% to 7.1%] |
| 2013 or later | 7 | 97.4% | 5.7% [1.8% to 8.4%] |
| Single centre vs multicentre studies |  |  |  |
| Single centre | 10 | 97.5% | 4.8% [1.5% to 6.4%] |
| Multicentre | 4 | 97.5% | 4.4% [1.7% to 7.7%] |
| Inclusion of ICU |  |  |  |
| Included | 2 | - | 5.1% [1.8% to 8.4%] |
| Excluded or not reported | 12 | 98.2% | 4.8% [1.5% to 6.7%] |
| Age groups (as reported) |  |  |  |
| Children | 4 | 93.4% | 1.2% [0.5% to 3.7%] |
| Adults | 9 | 96.1% | 6.2% [2.6% to 7.1%] |
| Adults and children | 1 | - | 7.0% [7.0% to 7.0%] |
| Age groups (splitting the dataset of Russom 2017) |  |  |  |
| Children | 5 | 97.6% | 1.8% [0.6% to 4.7%] |
| Adults | 10 | 96.6% | 6.3% [2.6% to 8.4%] |
| Study duration |  |  |  |
| <=1 month | 2 | - | 5.1% [1.8% to 8.4%] |
| >1 month to <=6 months | 5 | 92.4% | 6.2% [5.7% to 7.0%] |
| >6 months | 7 | 98.0% | 2.6% [0.6% to 6.4%] |
| ADR definition used |  |  |  |
| WHO | 9 | 98.3% | 5.7% [2.6% to 6.4%] |
| Aronson & Ferner / Edwards & Aronson | 3 | - | 1.8% [0.6% to 8.4%] |
| Not reported | 2 | - | 4.3% [1.5% to 7.1%] |
| Detection method |  |  |  |
| Active surveillance | 13 | 98.2% | 5.7% [1.8% to 7.0%] |
| Spontaneous reporting | 1 | - | 1.5% [1.5% to 1.5%] |
| Surveillance orientation (if active surveillance, n = 13) |  |  |  |
| Prospective | 9 | 97.6% | 5.7% [2.6% to 7.0%] |
| Retrospective | 2 | - | 6.8% [6.4% to 7.1%] |
| Pro- and retrospective | 2 | - | 1.1% [0.4% to 1.8%] |
| Surveillance team (if active surveillance, n = 13) |  |  |  |
| Single investigator | 2 | - | 8.0% [5.7% to 10.3%] |
| Multidisciplinary team | 7 | 99.0% | 1.8% [0.6% to 7.0%] |
| Not reported | 4 | 71.3% | 5.2% [3.3% to 6.8%] |
| Review methods (if active surveillance, n = 13) |  |  |  |
| Folder review alone | 9 | 93.9% | 6.2% [2.6% to 7.0%] |
| Folder review augmented by other methods | 4 | 98.9% | 2.3% [0.5% to 7.1%] |
| Causality assessment method |  |  |  |
| Naranjo | 5 | 94.5% | 7.0% [5.7% to 7.1%] |
| WHO-UMC | 4 | 95.9% | 5.1% [2.9% to 7.3%] |
| Other methods | 3 | - | 0.6% [0.4% to 2.6%] |
| Not reported or not done | 2 | - | 4.0% [1.5% to 6.4%] |
| Causal categories included in numerator |  |  |  |
| Definite, probable, and possible | 9 | 98.4% | 5.7% [1.8% to 7.0%] |
| Definite and probable | 2 | - | 6.4% [2.6% to 10.3%] |
| Not reported | 1 | - | 0.6% [0.6% to 0.6%] |

### Supplementary Figure S1. Proportion of admissions attributable to ADRs (Group 1 studies), by study quality


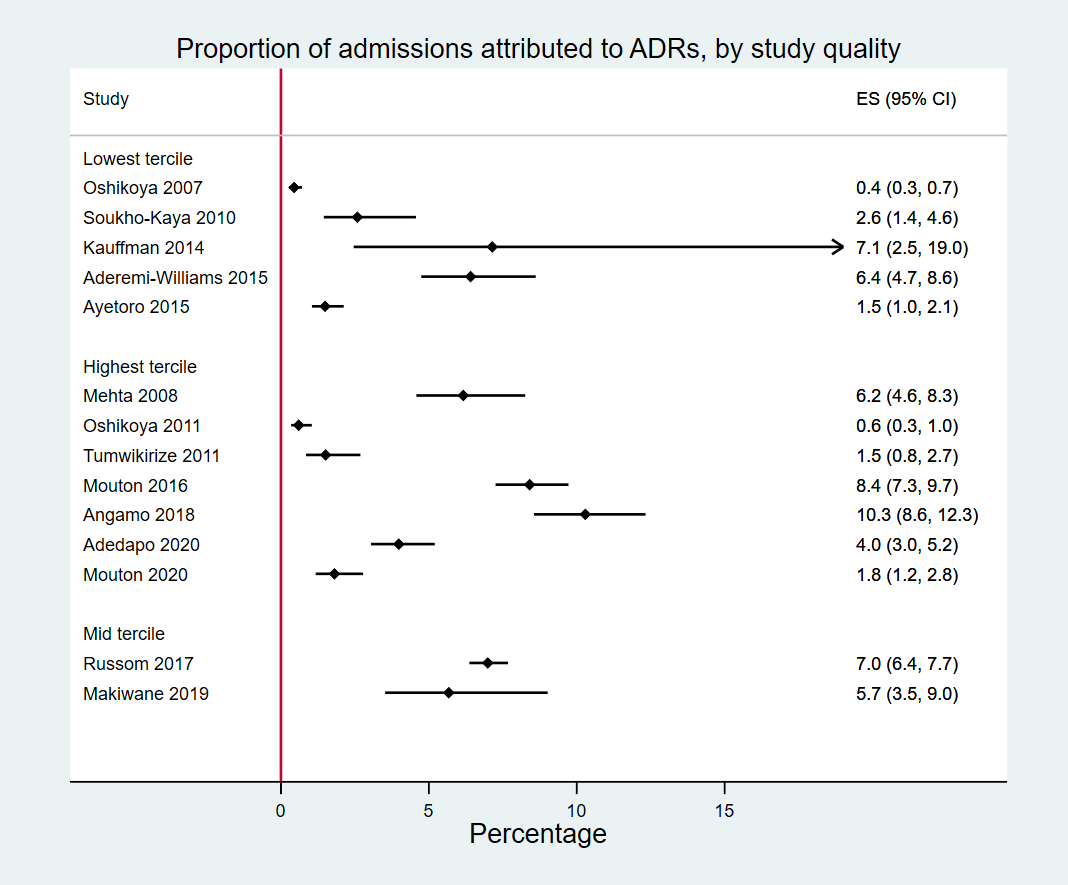


### Supplementary Figure S2. Proportion of admissions attributable to ADRs (Group 1 studies), by study setting


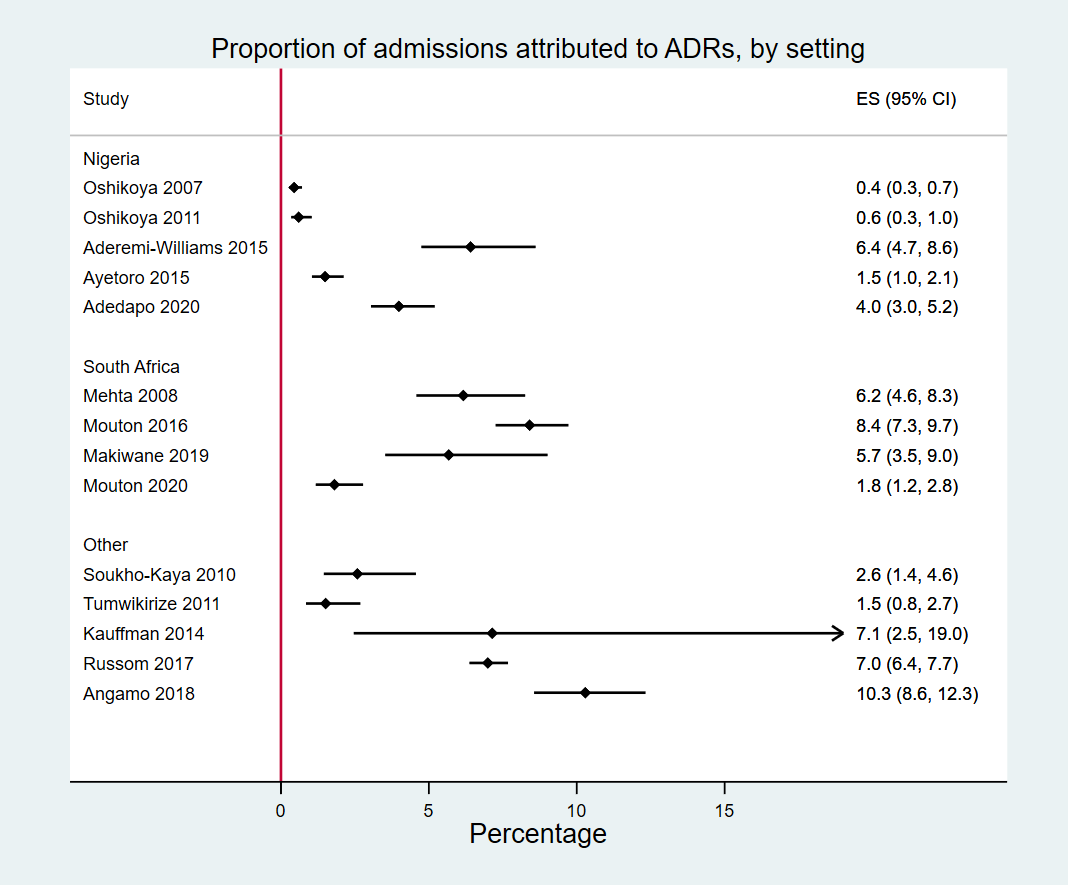


### Supplementary Figure S3. Proportion of admissions attributable to ADRs (Group 1 studies), by year of data collection


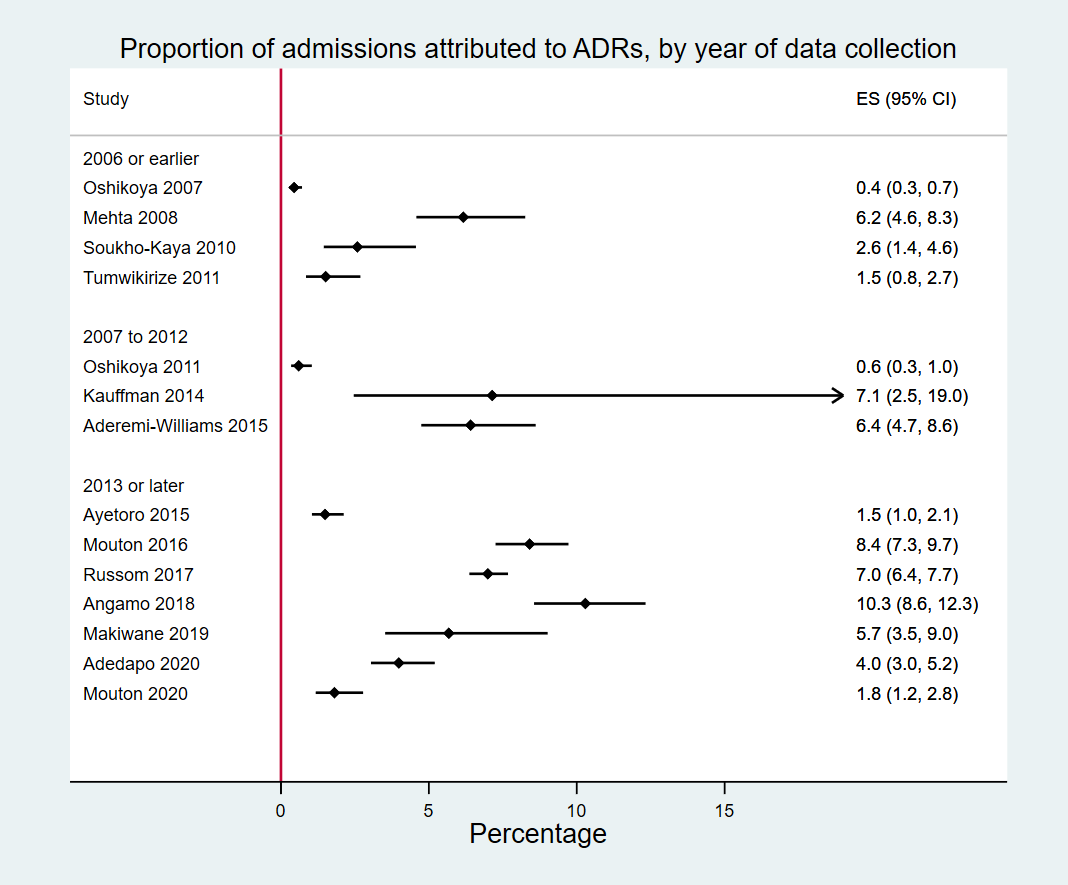


### Supplementary Figure S4. Proportion of admissions attributable to ADRs (Group 1 studies), by number of study centres


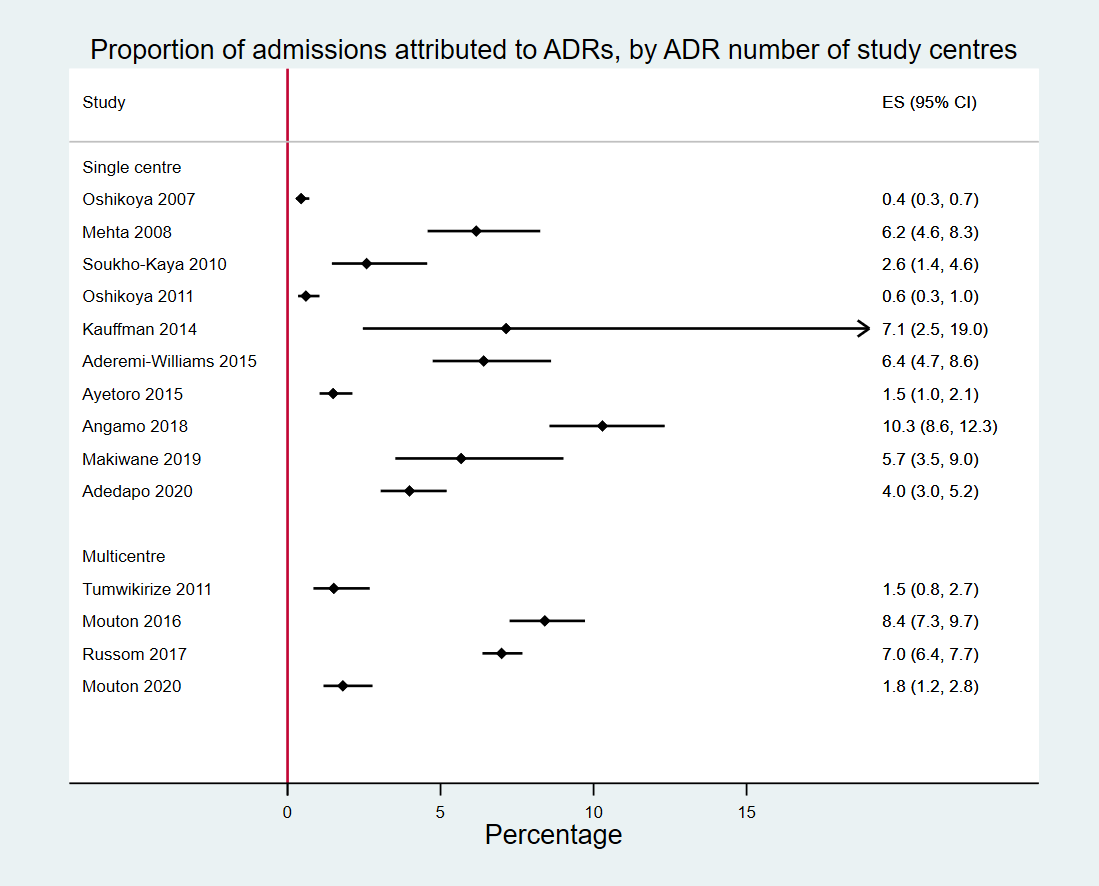


### Supplementary Figure S5. Proportion of admissions attributable to ADRs (Group 1 studies), by inclusion of intensive care units


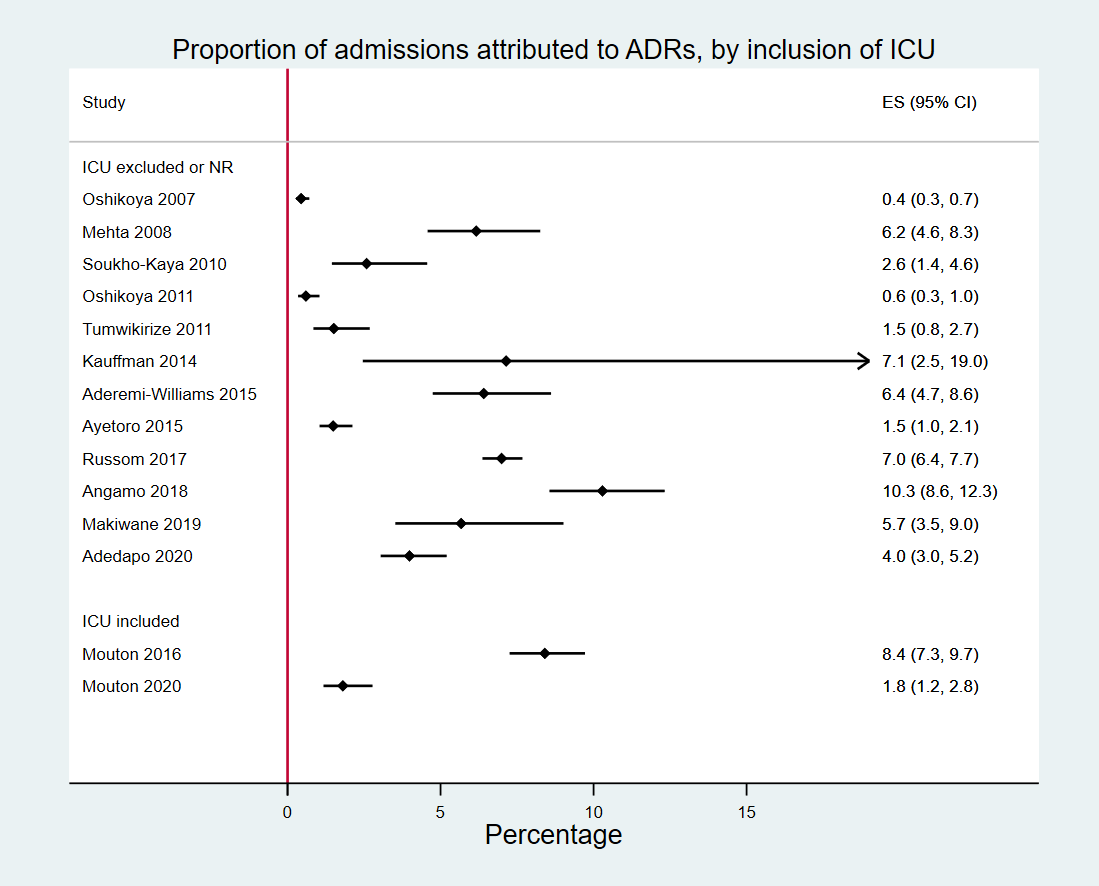


### Supplementary Figure S6. Proportion of admissions attributable to ADRs (Group 1 studies), by age groups (as reported)


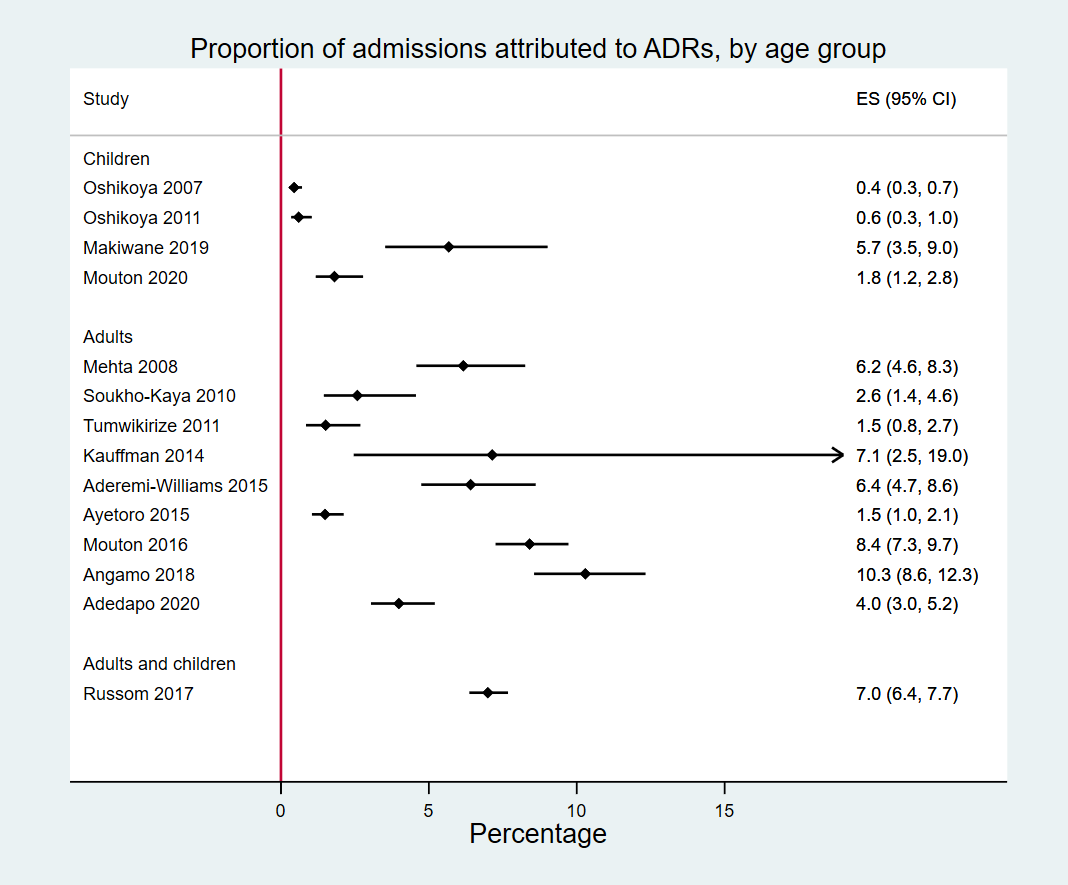


### Supplementary Figure S7. Proportion of admissions attributable to ADRs (Group 1 studies), by age groups (extracting adult and paediatric datasets from Russom 2017)


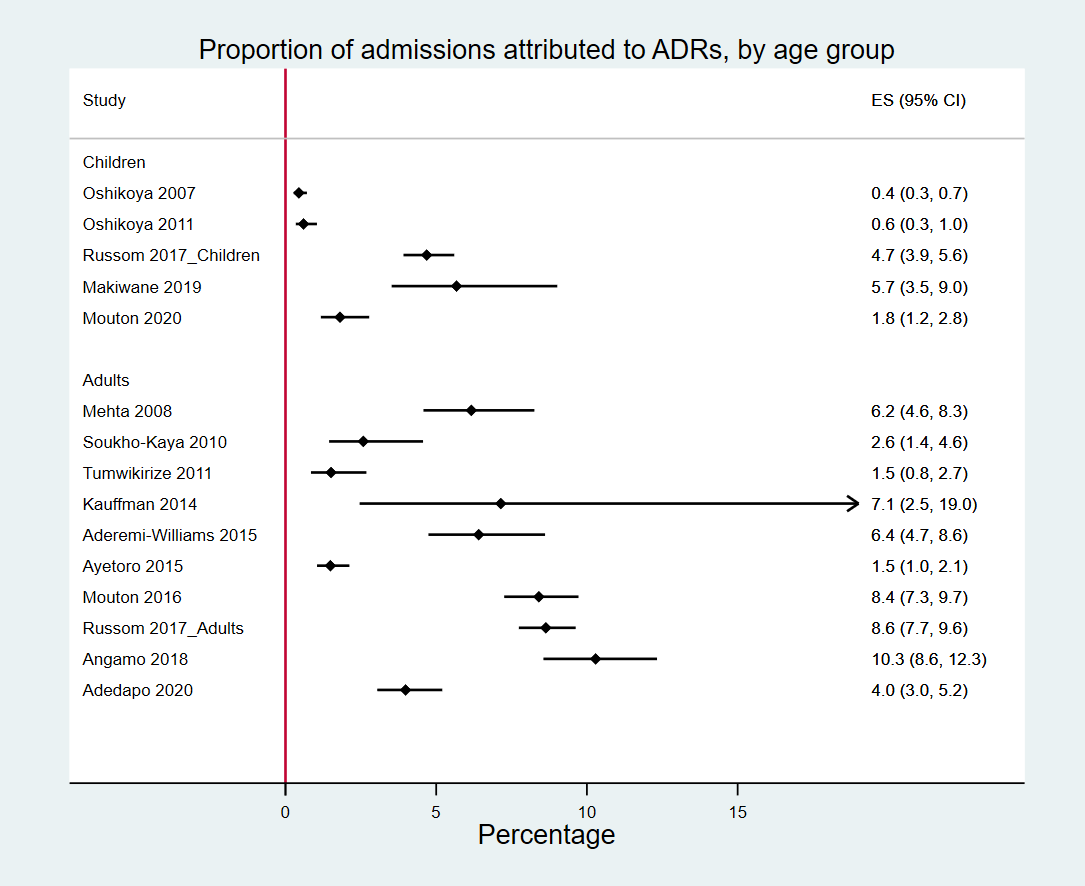


### Supplementary Figure S8. Proportion of admissions attributable to ADRs (Group 1 studies), by study duration


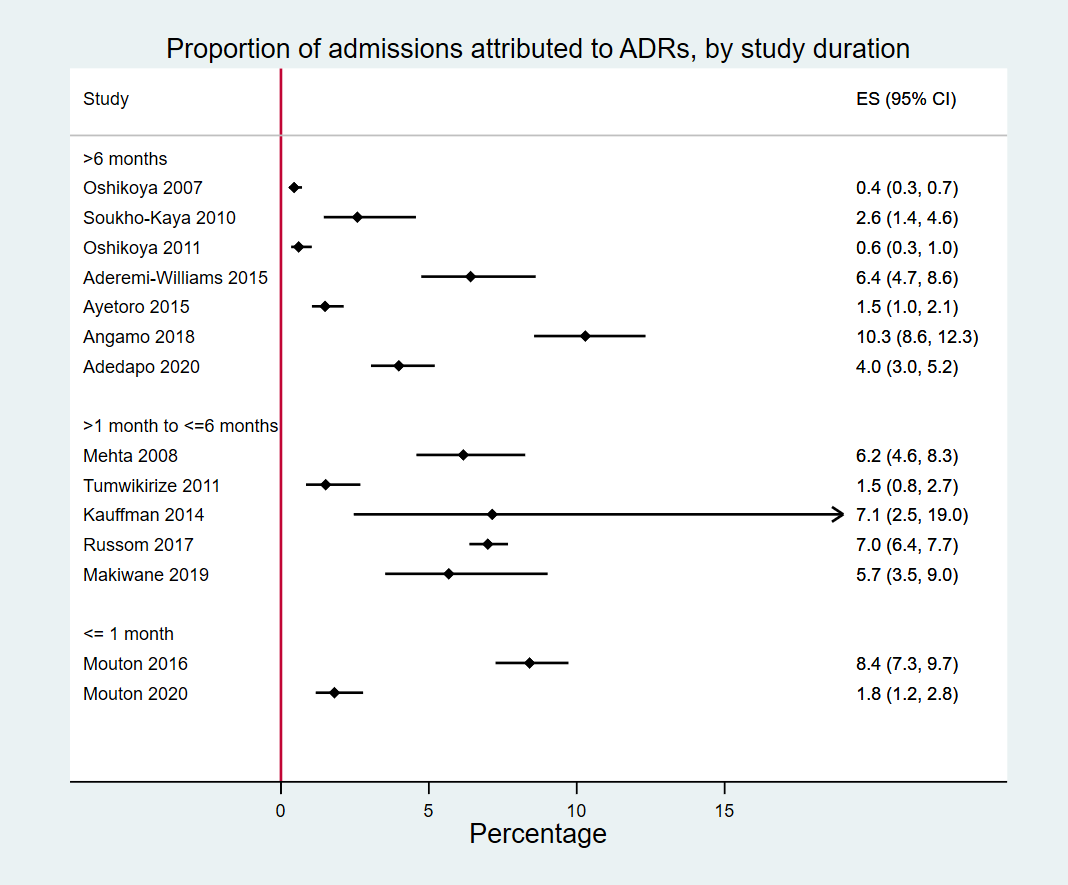


### Supplementary Figure S9. Proportion of admissions attributable to ADRs (Group 1 studies), by ADR definition


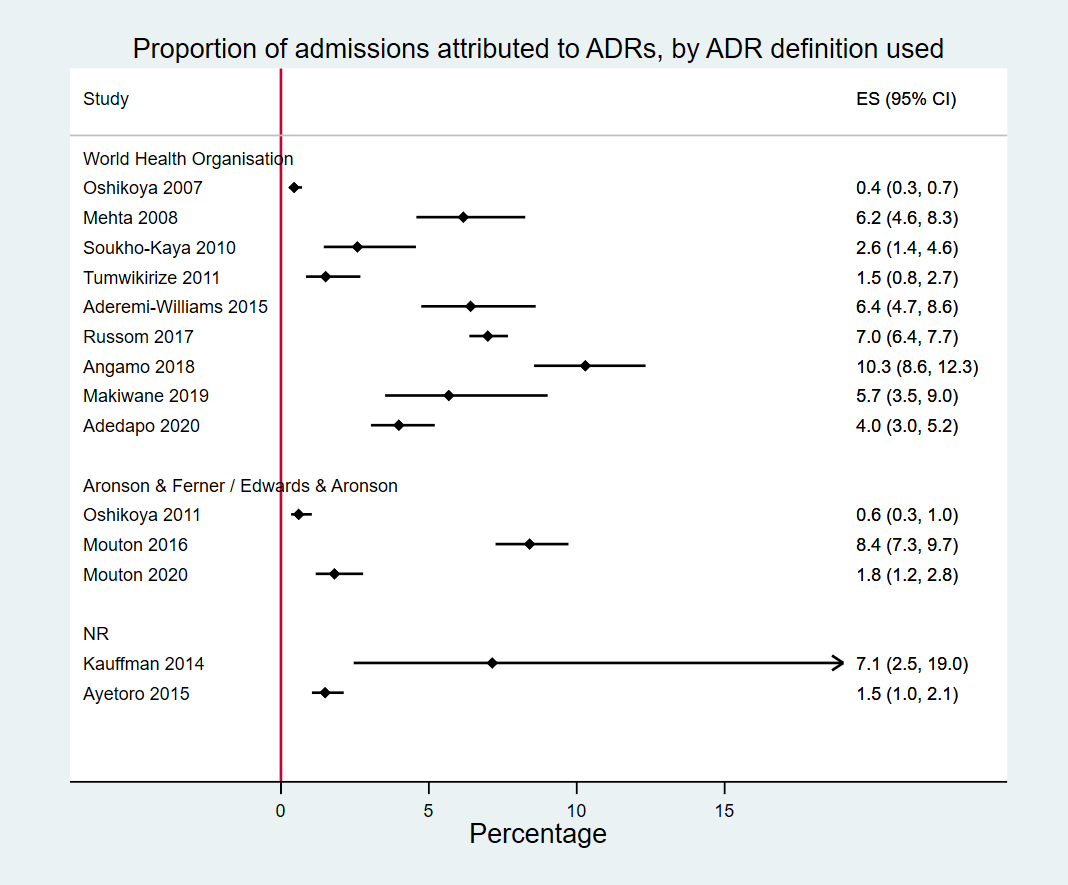


### Supplementary Figure S10. Proportion of admissions attributable to ADRs (Group 1 studies), by ADR detection method


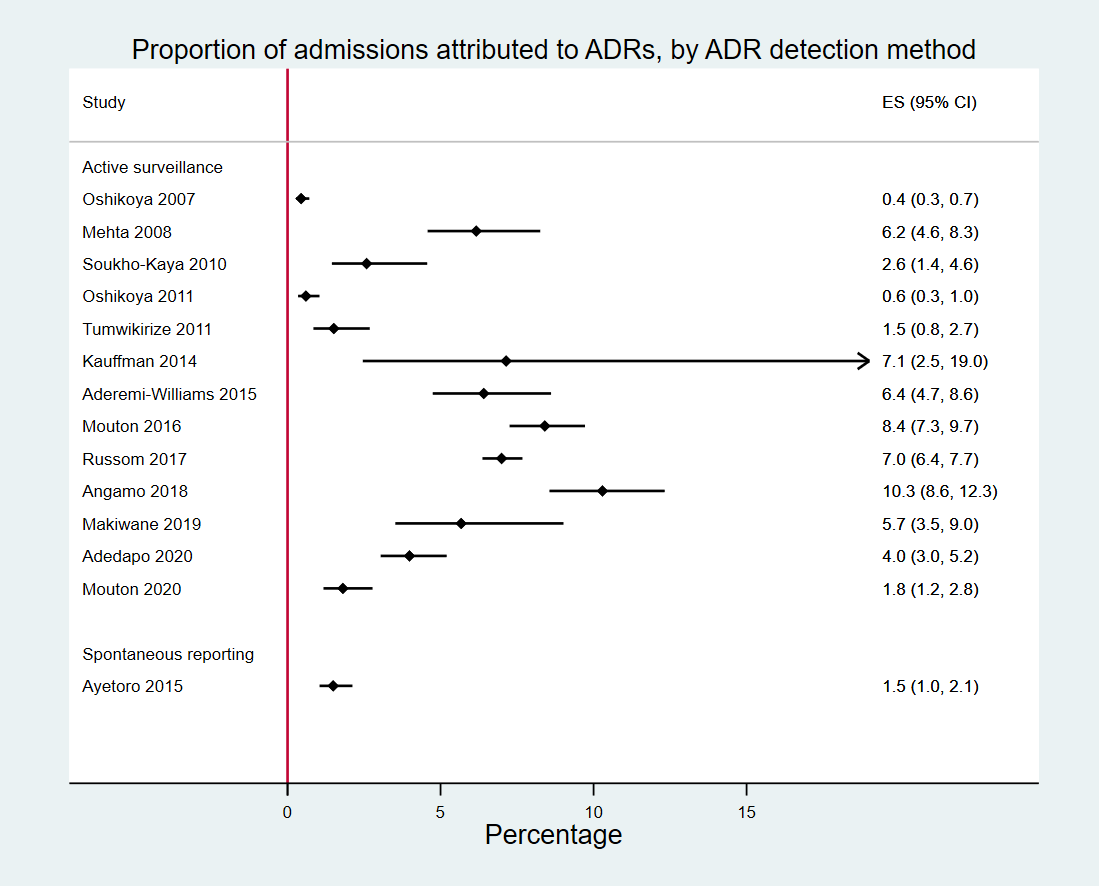


### Supplementary Figure S11. Proportion of admissions attributable to ADRs (Group 1 studies), by study orientation


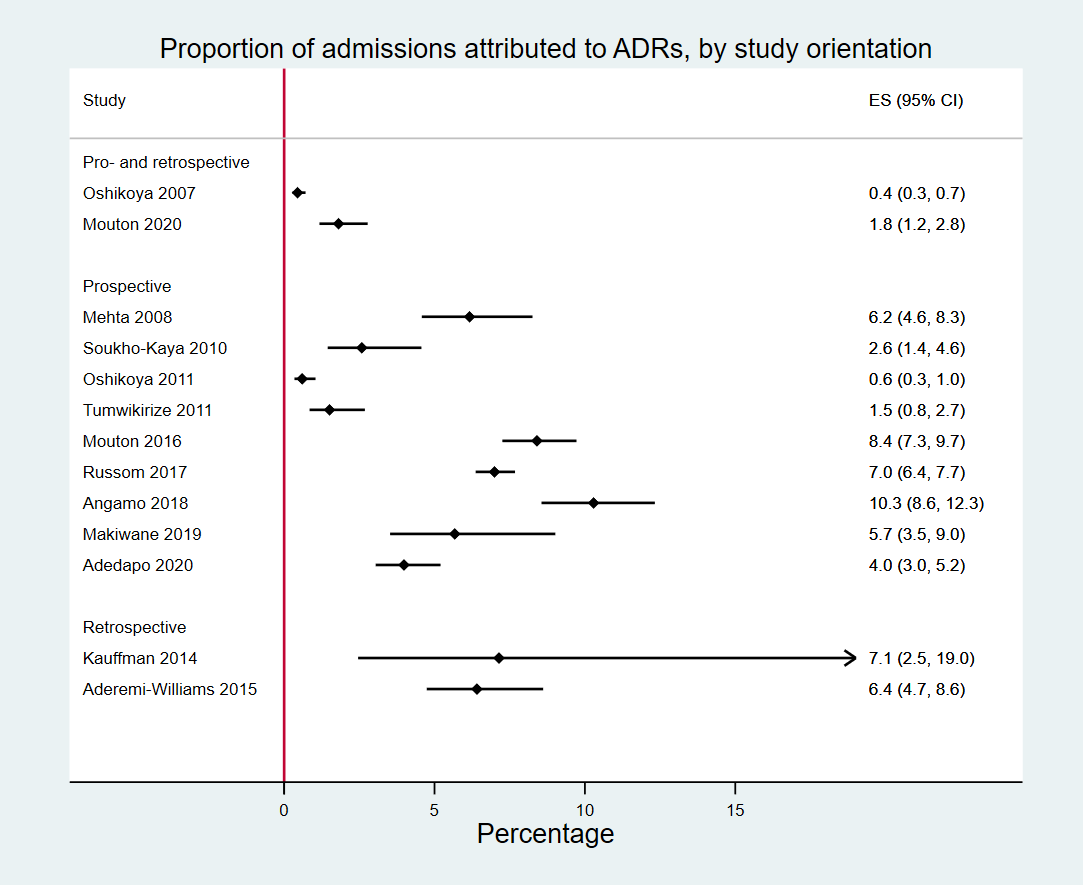


### Supplementary Figure S12. Proportion of admissions attributable to ADRs (Group 1 studies), by surveillance team


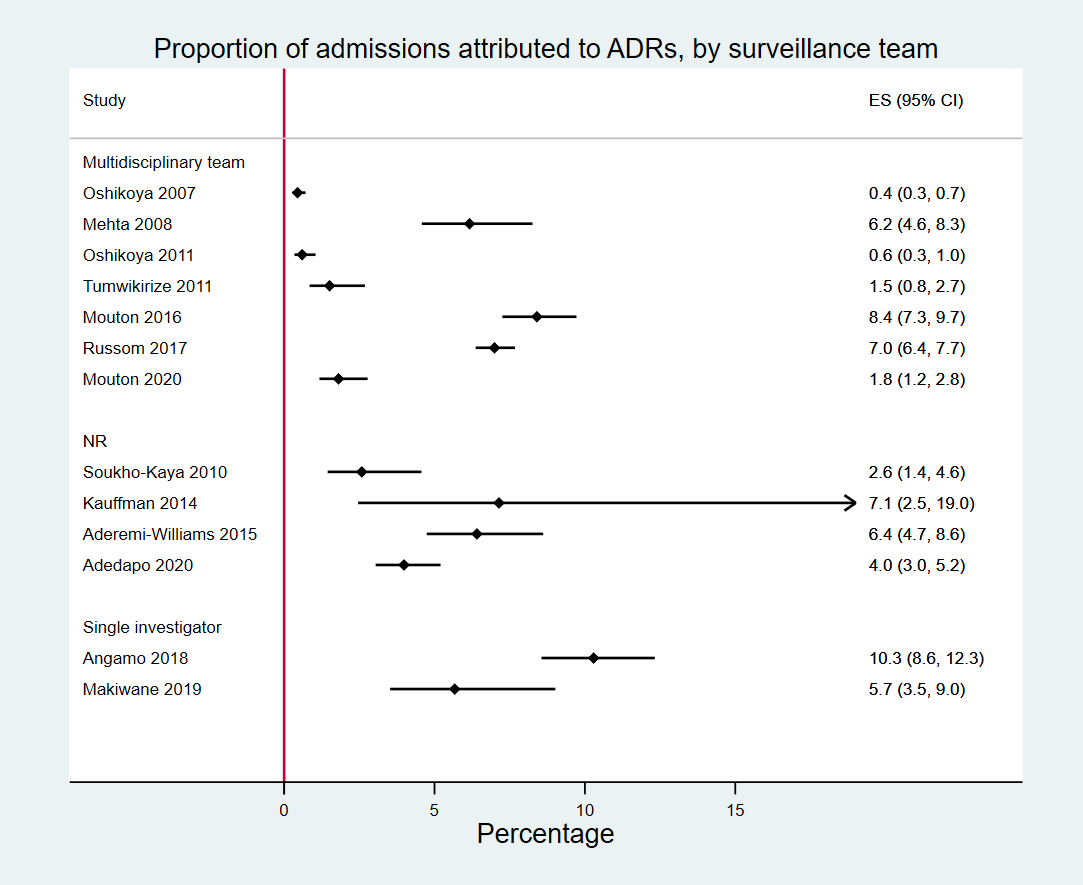


### Supplementary Figure S13. Proportion of admissions attributable to ADRs (Group 1 studies), by folder review method


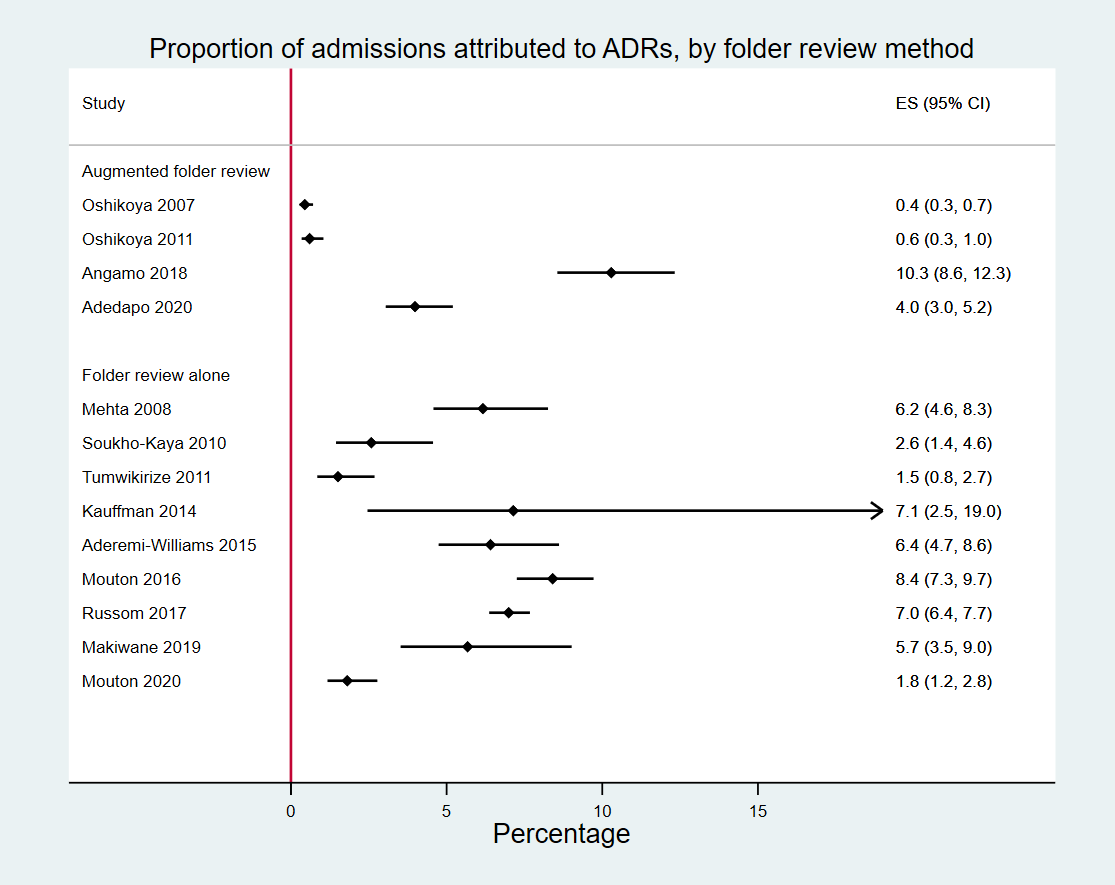


### Supplementary Figure S14. Proportion of admissions attributable to ADRs (Group 1 studies), by causality assessment method


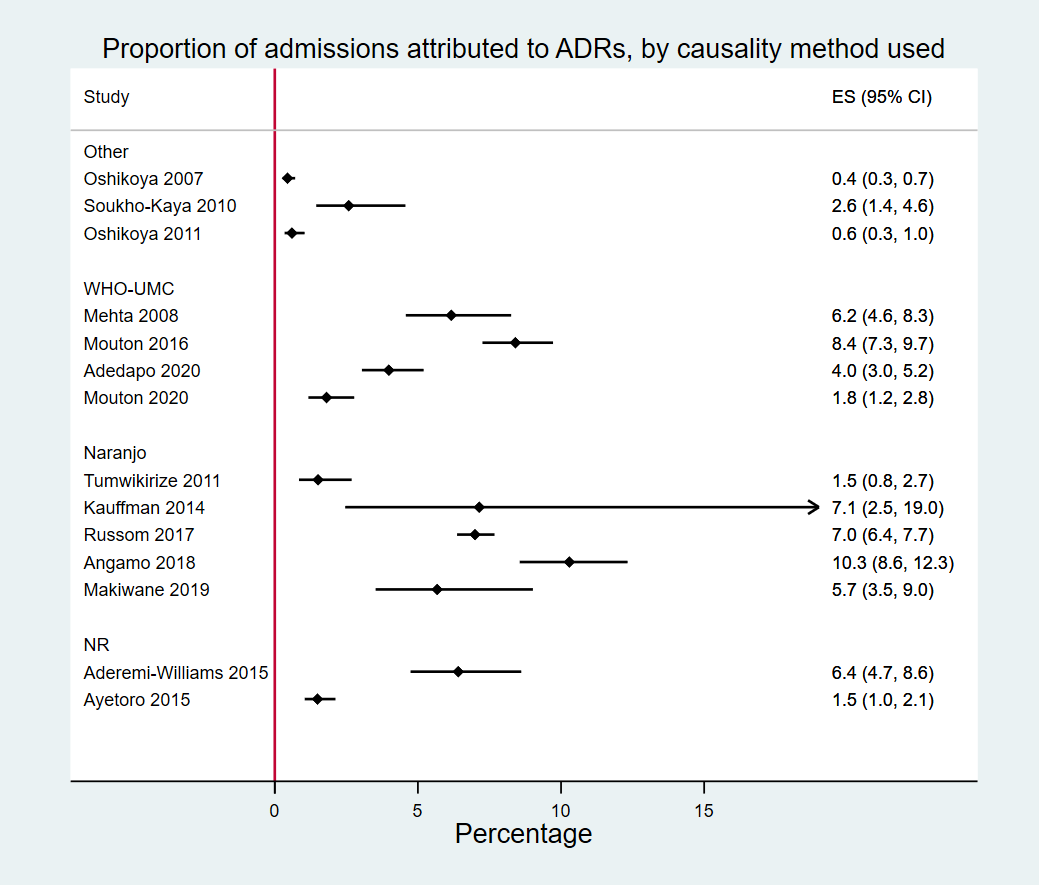


### Supplementary Figure S15. Proportion of admissions attributable to ADRs (Group 1 studies), by causality categories included


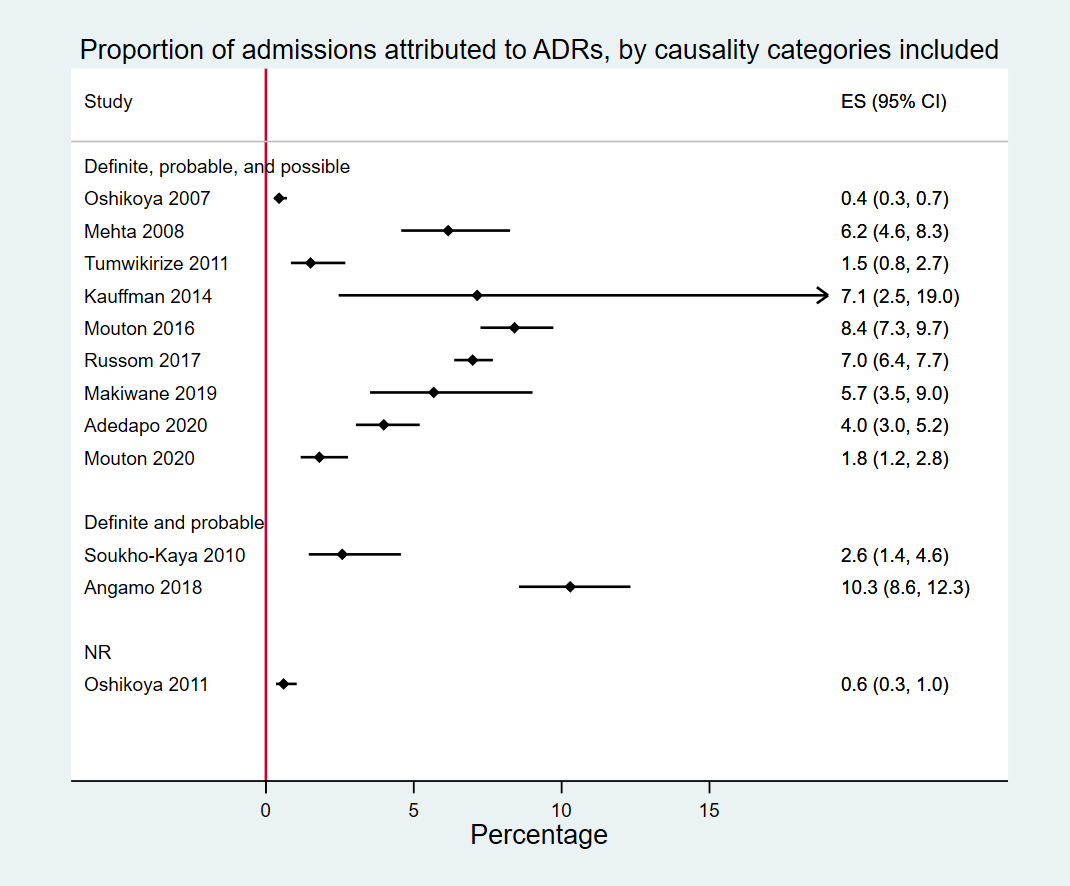


### Supplementary Figure S16. Proportion of admissions prolonged by ADRs (Group 2 studies)


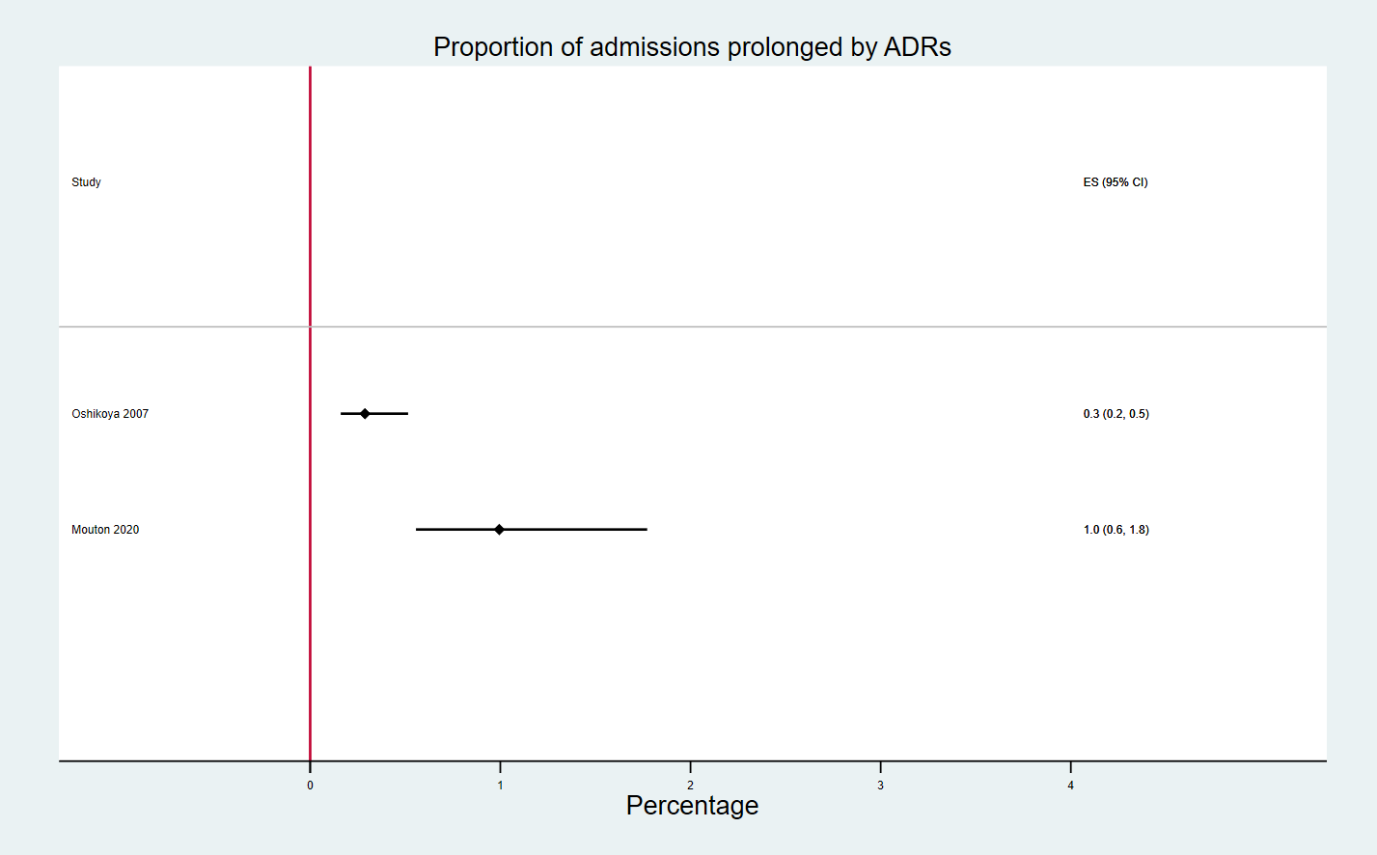


### Supplementary Figure S17. Proportion of in-hospital deaths attributable to ADRs (Group 3 studies)


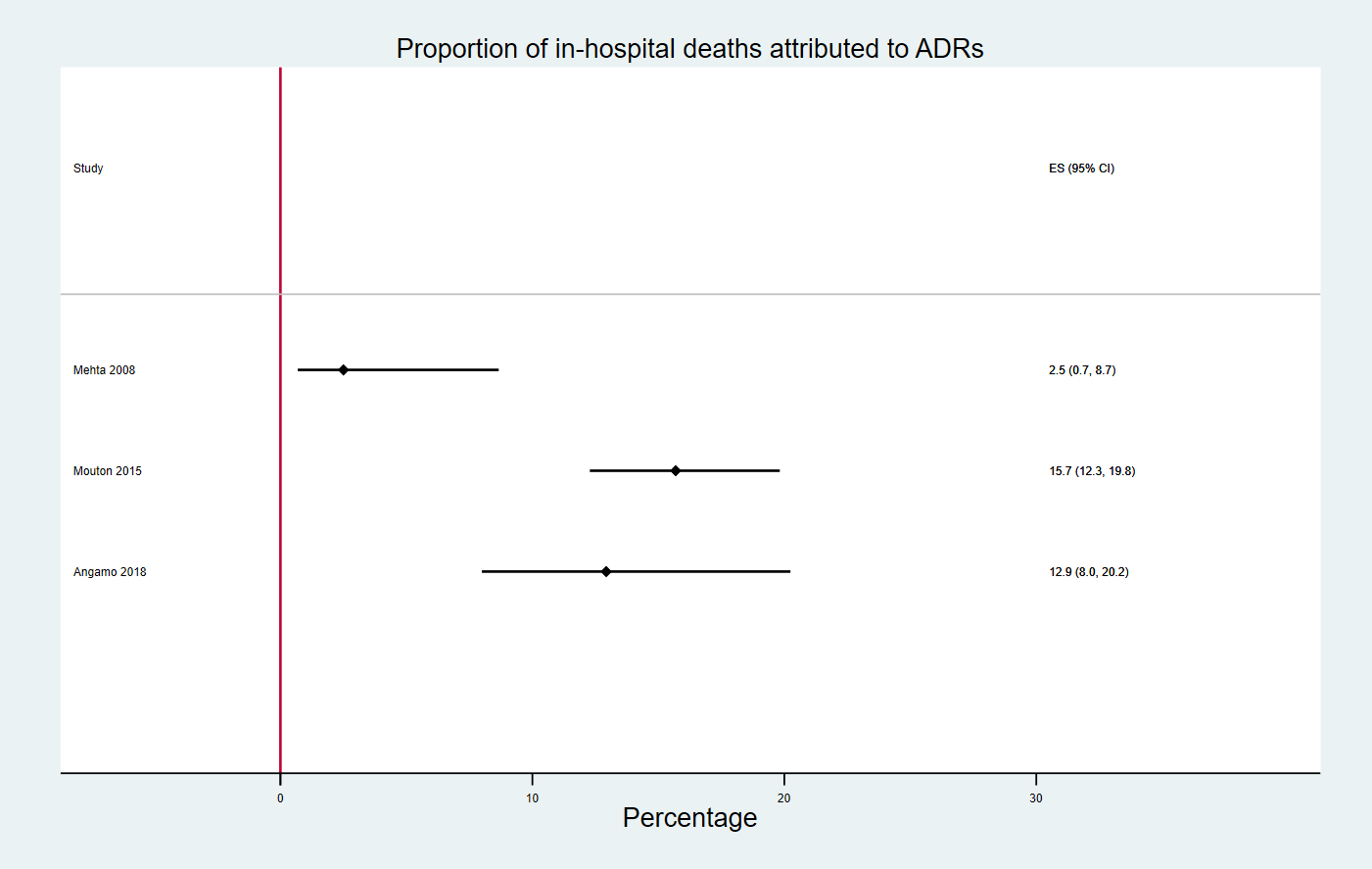

Supplement: Supplementary file 1 — Supplementary Material [file PRP2-9-e00875-s001.docx]
